# Association of *Porphyromonas gingivalis*-infected oral squamous cell carcinoma cell-secreted exosomal miR-3648-1-p5 with tumor progression

**DOI:** 10.1186/s12935-026-04230-5

**Published:** 2026-03-04

**Authors:** Xiaorong Tan, Zhangping Tan, Wenyue Xu, Yuchuan Zhou, Wei Wei, Chenxi Li, Muqiu Li, Zhongcheng Gong

**Affiliations:** 1 Oncological Department of Oral and Maxillofacial Surgery, the First Affiliated Hospital of Xinjiang Medical University, School / Hospital of Stomatology Xinjiang Medical University, Urumqi 830054, Xinjiang Uygur Autonomous Region China; 2 Stomatological Research Institute of Xinjiang Uygur Autonomous Region, Urumqi 830054, Xinjiang Uygur Autonomous Region China; 3 Department of Oncology, Chongqing University Affiliated Renji Hospital (Chongqing Fifth People’s Hospital), Chongqing, 404100 China; 4 Chongqing Center for Disease Control and Prevention, Chongqing 400707,, China; 5 Department of Pathogenic Biology, Army Medical University (Third Military Medical University), Chongqing 400038, China; 6 General Practice Medicine college of, XinJiang Medical University, Urumqi 830054, Xinjiang Uygur Autonomous Region, China

**Keywords:** Oral squamous cell carcinoma, Porphyromonas gingivalis, *P. gingivalis*-infected OSCC cell, Exosomes, miR-3648-1-p5

## Abstract

**Background:**

Oral squamous cell carcinoma accounts for over 90% of oral malignancies and remains a major global public health concern. Our previous study identified *Porphyromonas gingivalis* (*P. gingivalis*) as a key microbial factor in oral squamous cell carcinoma initiation and progression. However, the precise mechanisms by which *P. gingivalis* infection contributes to tumor progression remain unclear. Pathogens regulate exosome secretion during bacterial infection; therefore, this study aimed to investigate the role of exosomes from *P. gingivalis*-infected oral squamous cell carcinoma cells (Pg.Ex) in tumor progression.

**Methods:**

We isolated exosomes using differential centrifugation. To elucidate the mechanisms by which Pg.Ex promote oral squamous cell carcinoma progression, microRNA sequencing was performed on exosomes isolated from *P. gingivalis*-infected and uninfected cells. Furthermore, the biological roles of Pg.Ex and exosomal miR-3648-1-p5 in oral squamous cell carcinoma tumor growth were examined in vitro and in vivo.

**Results:**

We found that *P. gingivalis*-infected oral squamous cell carcinoma cells released lower concentrations and larger particle sizes of exosomes. Pg.Ex had lower levels of miR-3648-1-p5 and could be transferred to uninfected cells to promote the malignant phenotype of oral squamous cell carcinoma cells, in vitro and in vivo.

**Conclusions:**

These findings reveal an unknown connection between low exosomal miR-3648-1-p5 expression in *P. gingivalis*-infected oral squamous cell carcinoma cells and oral squamous cell carcinoma progression. Therefore, targeting Pg.Ex and miR-3648-1-p5 may present a promising therapeutic strategy for the treatment of oral squamous cell carcinoma.

**Supplementary Information:**

The online version contains supplementary material available at 10.1186/s12935-026-04230-5.

## Background

Oral squamous cell carcinoma (OSCC) is one of the most common cancers worldwide, accounting for over 90% of all oral malignancies [1]. Alcohol consumption, betel nut consumption, and human papillomavirus infection are well-established risk factors for OSCC [2]. However, approximately 15% of OSCC cases lack identifiable causes [3], highlighting the crucial need to identify pathogenic factors to prevent the progression of oral tumors.

Several studies have confirmed that chronic inflammation caused by bacteria is associated with the development, invasion, and metastasis of malignancies. Approximately 20% of malignancies are directly associated with chronic infections [4]. Given the oral cavity’s rich bacterial population, increasing attention has been directed toward the relationship between the oral microbiome and OSCC progression. *Porphyromonas gingivalis* (*P. gingivalis*) is a dominant bacterium in oral diseases and a key risk factor for OSCC. *P. gingivalis* contributes to OSCC development and progression by inducing chronic inflammation and modulating cell proliferation, apoptosis, migration, invasion, and angiogenesis [5]. In our previous study, we demonstrated that *P. gingivalis* contributed to the development and progression of oral cancer [6]. However, the underlying mechanisms remain unclear. Historically, research has primarily focused on tumor-promoting components secreted by bacteria, while the role of *P. gingivalis*-infected OSCC cells in cancer progression remains unclear.

Intercellular communication is fundamental to normal physiological functions and plays a critical role in tumor progression and metastasis. Information can be transmitted through either direct cell contact or signaling molecules secreted by cells. Over the past two decades, intercellular signal transduction through extracellular vesicles, including exosomes, has been proposed as the third method of intercellular communication [7, 8]. Exosomes express markers, such as CD9, D63, CD81, and Tumor Susceptibility Gene 101 (TSG101), and carry RNA, microRNAs (miRNAs), DNA, and proteins. Exosomes also have antigen presentation, immune activation, and suppressive effects [9–12]. Among the most important components of extracellular vesicles, miRNAs account for only 1–3% of the human genome. However, they regulate nearly 33% of human genes and are involved in almost all tumor-related processes, including proliferation, differentiation, apoptosis, metastasis, angiogenesis, immune response, and chemotherapy resistance [13–17].

Pathogens modulate exosome production and secretion by host cells during viral, bacterial, parasitic, or fungal infections [18]. Previous studies demonstrated that exosomes from bacteria-infected hosts can stimulate cytokine production in recipient cells, thereby enhancing innate immune responses against bacterial infections [19]. Colorectal cancer cells carrying *Fusobacterium nucleatum* (*F. nucleatum*) produced exosomes rich in miR-1246, miR-92b-3p, miR-27a-3p, and chemokine (C-X-C motif) ligand 16, which are absorbed by uninfected cells and lead to prometastatic behavior [20]. *F. nucleatum*-infected colorectal cancer cells secrete exosome-wrapped miR-122-5p, which activate the TGF-β1/Smad signaling pathway to promote metastasis [21]. However, no studies have explored the role of exosomes secreted by *P. gingivalis*-infected OSCC cells in oral cancer. Therefore, this study aimed to assess the concentration, size, and differential miRNA of exosomes during *P. gingivalis* infection and to investigate their involvement in OSCC progression and development. These findings showed that *P. gingivalis*-infected OSCC cells released lower concentrations and larger particle sizes of exosomes. We revealed an unknown connection between low exosomal miR-3648-1-p5 expression in *P. gingivalis*-infected OSCC cells and OSCC progression.

## Methods

### Ethics statement

All mandatory laboratory health and safety procedures have been complied with in the course of conducting the experimental work reported in this paper. The study was conducted in accordance with the Declaration of Helsinki and the Animal Research: Reporting of In Vivo Experiments (ARRIVE) 2.0 guidelines and approved by the Institutional Review Board of The First Affiliated Hospital of Xinjiang Medical University (approval number A230307).

### Cell lines and culture

This study involved four OSCC cell lines (SCC25, SCC9, Cal-27, and SAS). SCC25 and SCC9 cell lines were purchased from BNCC (Beijing, China) and authenticated using short tandem repeats genotype testing. Both are cultured in DMEM/F12 + 10% fetal bovine serum (FBS) + 1% penicillin/streptomycin + 400 ng mL^− 1^ hydrocortisone (Servicebio, Wuhan, China). Cal-27 and SAS cell lines were acquired from Meisen (Hangzhou, China) and cultured in DMEM/high glucose medium (Gibco, Grand Island, NY, USA) supplemented with 10% FBS (Gibco). All human OSCC cell lines were maintained in a humidified incubator at 37 °C with 5% CO_2_.

### Bacterial culture and OSCC cell infection

The *P. gingivalis* W83 strain and *Veillonella parvula* (*V. parvula*) were purchased from BNCC. It has been reported that *V. parvula* does not affect OSCC cell proliferation; therefore, it was used as a control bacterium for *P. gingivalis* [22]. *P. gingivalis* was cultured anaerobically at 37℃ for 10 days in a Columbia blood agar plate, while *V. parvula* was cultured under the same condition for 3 days. Both *P. gingivalis* and *V. parvula* were then grown anaerobically at 37 °C for 48 h in Columbia broth (Solarbio, Beijing, China) supplemented with 5% FBS. OSCC cells at approximately 80% confluence were infected with either *P. gingivalis* or *V. parvula* at a multiplicity of infection (MOI) of 100 for 6 h. *P. gingivalis* -infected OSCC cells were washed with phosphate-buffered saline (PBS) and treated with fresh medium containing gentamicin (0.5 mg mL^− 1^) and metronidazole (0.1 mg mL^− 1^) to eliminate external adherent bacteria [23, 24]. After 1 h, the *P.gingivalis*-infected OSCC cells were co-cultured in DMEM/F12 without FBS for 48 h. Subsequently, cell culture supernatants were collected for exosome isolation.

### Exosome isolation, purification, and identification

Exosomes were isolated using differential centrifugation [25]. Cell supernatants were sequentially centrifuged at 300 × *g* for 10 min, 3,000 × *g* for 15 min, and 10,000 × *g* for 30 min to remove cells and large debris. The clarified supernatant was then filtered through a 0.22 μm membrane, followed by ultracentrifugation at 100,000 × *g* for 70 min at 4 °C. The resulting exosome granules were washed with PBS, and the process was repeated. Lastly, the pellet was resuspended in 100 µl of PBS to obtain purified exosomes, which were stored at − 80℃. Exosomes were divided into three groups: exosomes from non-infected OSCC cells (Ex), exosomes from *V. parvula*-infected OSCC cells (Vp.Ex), and exosomes from *P. gingivalis*-infected OSCC cells (Pg.Ex). The concentration and particle size distribution of exosomes were measured using a ZetaView Nanoparticle Tracking Analyzer PMX-120 (Particle Metrix, Meerbusch, Germany) equipped with a blue laser (5/40 mW, 488 nm). Data on particle density, size, and concentration distribution were obtained using NanoFCM software (NF Professional v1.08; Xiamen Fuliu Biotechnology Co. Ltd., Xiamen, China). For morphological analysis, purified exosomes were fixed in an equal volume of 4% paraformaldehyde for 10 min. Exosomes were then chemically stained with 1% uranyl acetate, and their ultrastructure was visualized using transmission electron microscopy (HT-7700; Hitachi, Marunouchi, Japan).

### Western blotting

Total exosomal proteins were abstracted using an exosome protein isolation kit (Bestbio, Shanghai, China), according to the manufacturer’s protocol. Proteins from SCC25 cells were extracted by centrifugation at 12,000 rpm for 10 min using RIPA lysis buffer (Servicebio). A BCA kit (1Use Biomedicine Co. Ltd., Guangdong, China) was used to measure the protein concentration in the supernatant and exosomes. Cells and exosomal proteins were separated using 10% SDS-PAGE, transferred to polyvinylidene fluoride membranes, and detected using antibodies against CD9 (1:1,000; Abcam, Cambridge, UK), CD63 (1:1,000; Abcam) [26]. Another exosomal marker was separated by 8% SDS-PAGE and detected using antibodies against TSG101 (1:1,000; Servicebio). Horseradish peroxidase-conjugated anti-rabbit antibodies (1:5,000; Beyotime, Shanghai, China) were used as secondary antibodies. β-Tubulin levels were determined using specific antibodies (1:1,000; Cell Signaling Technology, Boston, MA, USA) and served as a loading control.

### PKH26 staining for exosomes

Exosomes were labeled with PKH26 red fluorescent cell linker kits (MINI26-1KT; Sigma, St. Louis, MI, USA), following established guidelines [26]. Labeled exosomes were diluted with PBS and centrifuged at 100,000 × *g* for 70 min at 4 °C to remove excess dye, then resuspended in an equal volume of PBS. For exosome tracking, laser confocal microscopy was used to detect the time of exosome uptake by OSCC cells. SCC25 cells were cultured with PKH26-labeled exosomes (15 µg mL^− 1^) for 0, 6, 12, and 24 h. The nucleus of SCC25 cells was stained with DAPI (Servicebio) and imaged using a CLSM (NIKON Eclipse Ti, Tokyo, Japan).

### Cell counting kit-8 (CCK-8) assay

OSCC cells (3 × 10^3^ cells per well) were seeded into 96-well plates and cultured with one of the following substances for 0, 24, 48, or 72 h: *P. gingivalis* (MOI = 1, 10, 50, or 100), *P. gingivalis* (MOI = 100), or *V. parvula* (MOI = 100). For exosome co-culture, exosomes were quantified using a BCA assay kit. OSCC cells were co-cultured with PBS, Ex, Vp.Ex, and Pg.Ex for 0, 24, 48, and 72 h. Cell proliferation was detected by measuring the absorbance of each sample using CCK-8 (Servicebio) at a wavelength of 450 nm on an enzyme-linked immunosorbent assay detector (BIO-DL, Shanghai, China).

### Scratch-wound assay

The scratch-wound test was used to evaluate the migratory ability of OSCC cells. 3.5 × 10^4^ OSCC cells in 70 µl DMEM/F12 medium (Gibco) with 10% FBS were seeded into each well of an Ibidi Culture-Insert 2 Well (Ibidi, Martinsried, Germany) placed in 12-well plates. After cell adhesion, cells were infected with *P. gingivalis* or *V. parvula* for 6 h. Upon reaching full confluence, the culture insert was removed, creating a cell-free gap of approximately 500 μm. After washing with PBS, 1 mL DMEM/F12 without FBS was inoculated into a 12-well plate, and images of each treatment group were acquired using a microscope (Leica DM IL LED, Wetzlar, Germany) at 0 and 48 h after scratch formation. For exosome co-culture, the first image of the scratch was acquired using a microscope after forming a cell-free band of approximately 500 μm. The cells were then co-cultured with Ex, Vp.Ex, and Pg.Ex at a concentration of 15 µg mL^− 1^ for 48 h before the second image was acquired. The percentage of wound closure was calculated using the following formula: wound closure (%) = migrated cell surface area / total surface area × 100.

### Matrigel invasion assay

To assess the invasive capacity of the OSCC cells, OSCC cells were infected with *P. gingivalis* or *V. parvula* for 6 h and then seeded (4 × 10^4^) in the upper chambers of the Transwell inserts precoated with 50 µl of Matrigel (Corning Inc., Corning, NY, USA). For exosome co-culture, OSCC cells were pretreated with DMEM/F12 + 10% FBS containing 15 µg mL^− 1^ Ex, Pg.Ex, or Vp.Ex. After 48 h, co-cultured OSCC cells were seeded into the upper layer of the Transwell chamber with DMEM/F12 without serum. The lower chamber was filled with DMEM/F12 supplemented with 10% FBS and incubated for 48 h. Cells that migrated through the membrane were fixed with 4% paraformaldehyde and stained with 1% crystal violet. Five random fields were selected in each chamber and the number of invaded cells counted.

### MiRNA library construction and sequencing

Total RNA from the exosomes was used for miRNA library preparation and sequencing, conducted by LC-BIO Technology Co. Ltd. (Hangzhou, China). The RNA quantity and purity of each group were evaluated using a NanoDrop ND-1,000 (NanoDrop, Wilmington, DE, USA). RNA fragment integrity was evaluated using a Bioanalyzer 2,100 (Agilent, Santa Clara, CA, USA). Small RNAs with nucleotides ranging from 18 to 26 bp were used for library preparation. PCR products of the small RNAs were sequenced on an Illumina HiSeq 2 500 platform (Illumina, San Diego, CA, USA). Differentially expressed genes were identified using the DESeq2 package in R (version 3.6.0; Tencent Cloud, Shenzhen, China). The detailed methods and procedures of the small RNA experimental procedure, bioinformatics analysis, and analysis of differentially expressed miRNAs are presented in Additional File 1.

### qRT-PCR analysis

Total RNA was extracted from exosomes using a total exosome RNA isolation kit (GeneCreate Biotech Co. Ltd‌., Wuhan, China), following the manufacturer’s instructions. cDNA was synthesized using the Mir-X miRNA First-Strand Synthesis and TB Green qRT-PCR User Manual Kit (TaKaRa, Tokyo, Japan). The miRNA level was quantified using TB Green Premix Ex Taq II (Tli RNaseH Plus; TaKaRa) and analyzed using CFX96 Manager (Bio-Rad, Hercules, CA, USA). Furthermore, data were normalized to U6 small nuclear RNA expression and calculated using the 2^–△△CT^ method. The qPCR primers used are listed in Additional File 2: Table S1.

### MiRNA transfection

The miR-3648-1-p5 mimics, inhibitors, and negative control (NC) were designed and cloned by GenePharma (Shanghai, China). Following the manufacturer’s protocol, Lipofectamine 3,000 reagent (Invitrogen, Carlsbad, CA, USA) was used for transfection with miR-3648-1-p5 mimics, inhibitors, or NC oligonucleotides at a final concentration of 50 nM (Table 1).

**Table 1:** Sequences of the MicroRNA mimics and inhibitors

Name	Sequence (5’-3’)
miR-3648-1-p5 mimics	S: AUCGCCGAGGGCCGGUCG AS: ACCGGCCCUCGGCGAUUU
miR-3648-1-p5 inhibitors	CGACCGGCCCUCGGCGAU
Negative control	S: UUCUCCGAACGUGUCACGUTT
	AS: ACGUGACACGUUCGGAGAATT
Inhibitors N.C	CAGUACUUUUGUGUAGUACAA

AS, antisense; S, sense; NC, Negative control

### Animal models

Five-week-old female BALB/c nude mice (16–18 g without specific pathogens) were obtained from Beijing Vital River Laboratory Animal Technology Co. Ltd. (Beijing, China) and used for in vivo assays. All mice were housed in laminar flow cabinets under a 12-h light/dark cycle at 22 ± 2°C and a humidity of 44–55%. The mice were fed disinfected dry food and drinking water. To establish the xenograft model of human OSCC, SCC25 cells (5 × 10^6^) were resuspended with 100 µL PBS and injected subcutaneously into the flanks of BALB/c mice (*n* = 25). After 7 days, when tumors grew to approximately 100 mm^3^, the mice were randomly divided into five groups (*n* = 5 per group) and injected intratumorally with PBS, Ex, exosomes secreted by OSCC cells transfected with miR-3648-1-p5 mimics (Mimics.Ex), Pg.Ex, or exosomes secreted by OSCC cells transfected with miR-3648-1-p5 inhibitors (Inhibitors.Ex) (10 µg per time) every other day for 7 days. The body weight and tumor size were measured every 2 days. Tumor volumes were calculated using the formula: (length × width^2^) / 2. All mice were anesthetized and euthanized 1-week post-injection according to the American Veterinary Medical Association (AVMA) Guidelines for the Euthanasia of Animals (2020). Next, tumor tissues were isolated for histological examination. The in vivo experiment was designed with reference to the previous study [20].

### Tissue preparation and hematoxylin–eosin and immunohistochemistry staining

Following standard protocols, harvested tumor tissues were fixed in 10% formalin for 24 h, processed, and embedded in paraffin. Sections of 5-µm thickness were then dewaxed, rehydrated, and rinsed. The sections were stained using immunohistochemistry or hematoxylin–eosin [27]. Immunohistochemistry was performed to assess the expression of Ki-67 in xenograft tumors using an anti-Ki-67 rabbit polyclonal antibody (1:200) (pPA5-114437). The tissues were then washed three times with PBS and incubated with an anti-rabbit secondary antibody (1:200) (GB23303; Servicebio) at 37 °C for 50 min. Following additional rinsing, the signal was developed by incubating the section with 3-diaminobenzidine tetrahydrochloride (Servicebio) for 10 s, followed by counterstaining with 10% Mayer’s hematoxylin. Stained tissue sections were scanned under a microscope (Olympus Corporation, Tokyo, Japan) at 20× magnification, and the percentage of stained cells was quantified in five randomly selected fields for each histological image for statistical analysis.

### Statistical analysis and reproducibility

Unless stated otherwise, data are expressed as mean (SD). A two-tailed unpaired Student’s *t*-test was used to compare two independent datasets. One- or two-way analysis of variance (ANOVA) was used to compare the means of multiple groups. Statistical significance was set at *P* < 0.05. All statistical analyses were conducted using GraphPad Prism (version 8.0) (San Diego, CA, USA).

## Results

### Screening and validating oral cancer cell lines with more active malignant behaviors

**Fig. 1:**
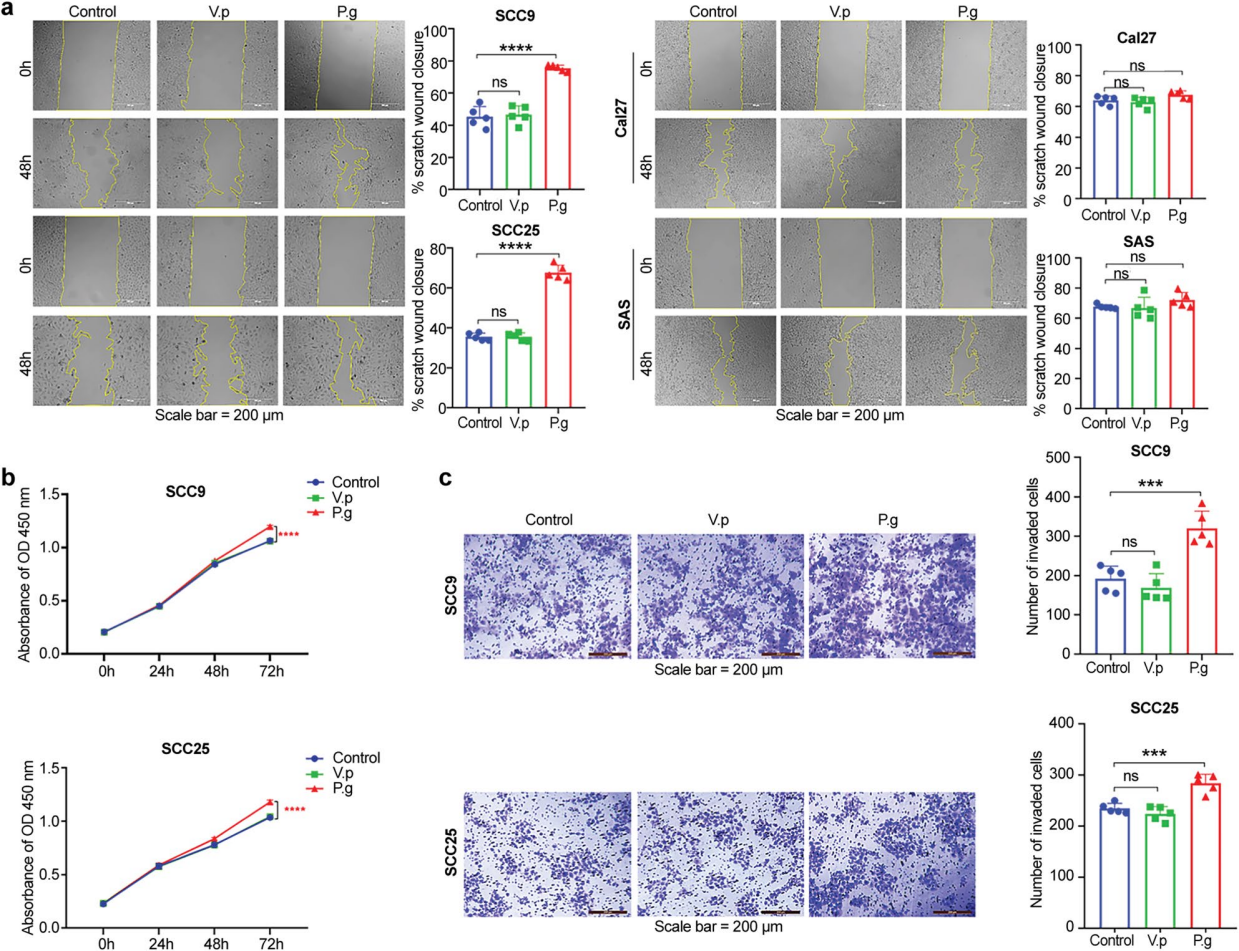
SCC25 and SCC9 cells exhibit more active malignant behavior than the other cells. **(a)** Migration of SCC25, SCC9, Cal-27, and SAS cells incubated with *P. gingivalis* or *V. parvula*. **(b)** and **(c)** Proliferation and invasion of SCC25 and SCC9 cells stimulated with *P. gingivalis* or *V. parvula* (MOI = 100). Scale bar, 200 μm. Data are presented as mean (SD). ns, not significant; ***P < 0.001; ****P < 0.0001. Data in **(a)** are analyzed using two-way ANOVA and those in **(b)** and **(c)** using one-way ANOVA

The scratch wound healing assay showed that *P. gingivalis* with an MOI of 50, 100, and 150 was more effective in promoting OSCC cell migration than that with an MOI of 1 and 10 (Additional File 2: Figure S1a). Furthermore, the CCK-8 assay demonstrated that *P. gingivalis* infection promoted OSCC cell proliferation, and an MOI of 100 and 150 exhibited greater effects than an MOI of 1, 10, and 50 (Additional File 2: Figure S1b). Therefore, all coculture experiments were conducted at an MOI of 100 for 48 h. Owing to the varying tumor-promoting effects among *P. gingivalis*-infected OSCC cell lines, we compared the migration of SCC25, SCC9, Cal-27, and SAS cells incubated with *P. gingivalis* or *V. parvula*. The results showed that *P. gingivalis* significantly enhanced the migration of SCC25 and SCC9 cells (*P* < 0.0001) compared to the control and *V. parvula* groups, whereas Cal-27 and SAS cells exhibited only a slight non-significant migration (Fig. 1a). The CCK-8 assay further confirmed that *P.gingivalis*-infected SCC25 and SCC9 cells exhibited greater proliferative capacity than control and *V. parvula* -treated cells (Fig. 1b). Additionally, transwell assays indicated that the invasion of SCC25 and SCC9 cells incubated with *P. gingivalis* was significantly higher than that of the control and *V. parvula* treatment groups (compared with control, *P* = 0.0003, *P* = 0.0003; Fig. 1c). These results show that *P. gingivalis* induces the malignant phenotype of SCC25 and SCC9 cells.

### *P. gingivalis* infection decreases exosome release from OSCC cells

**Fig. 2:**
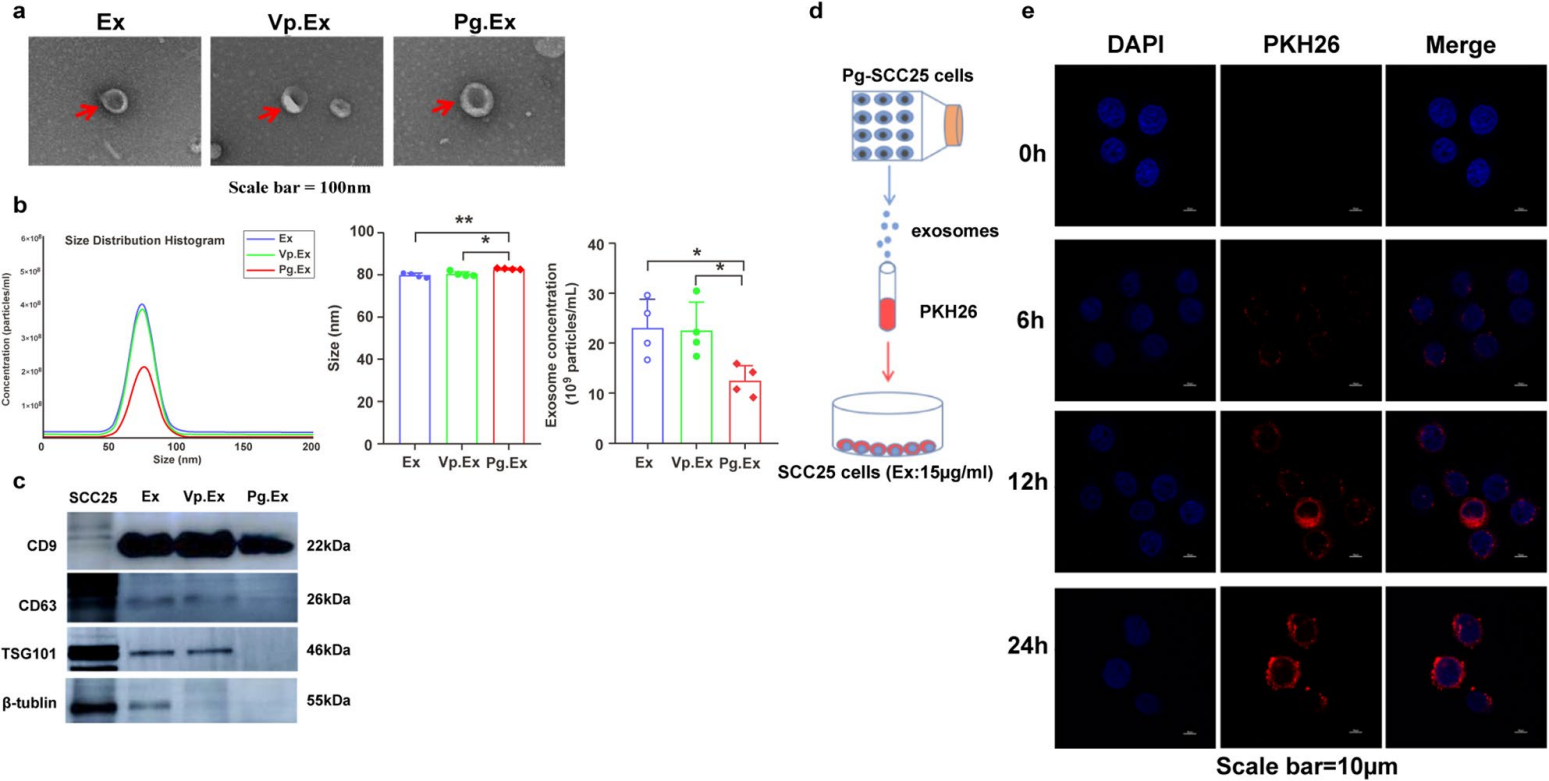
Verification of exosomes released from Ex, Vp.Ex, and Pg.Ex. **(a)** Transmission electron microscopy images of Ex, Vp.Ex, and Pg.Ex. Scale bar, 100 nm. **(b)** Nanoparticle Tracking Analyzer concentration and size in Ex, Vp.Ex, and Pg.Ex mice (*n* = 4). **(c)** Western blot analysis of exosomal markers (CD9, CD63, and TSG101) in Ex, Vp.Ex, and Pg.Ex, with β-tubulin used as the internal control. **(d)** Experimental design of exosome tracking. SCC25 cells (5 × 10^4^) are co-cultured with PKH26-labeled Pg.Ex (15 µg mL^− 1^) for different periods. **(e)** Confocal microscope images of SCC25 cell uptake of fluorescent staining. Scale bar, 10 μm. Data are presented as mean (SD) in **b)** and **c)**. *P < 0.05; **P < 0.01. Data in **b)** are analyzed using one-way ANOVA

To investigate the specific exosomes extracted from *P. gingivalis*-infected OSCC cells, exosomes were divided into three groups: Ex, Vp.Ex, and Pg.Ex. Ex and Vp.Ex were used as Pg.Ex controls. The transmission electron microscopy results revealed that the exosomes had elliptical and spherical shapes (Fig. 2a). Nanoparticle tracking analysis revealed that Pg.Ex exhibited lower particle concentration and larger size compared to Ex and Vp.Ex (compared with Ex, *P* = 0.0369, *P* = 0.0028; compared with Vp.Ex, *P* = 0.046, *P* = 0.013; Fig. 2b), whereas Ex and Vp.Ex exhibited similar characteristics. Western blot analysis confirmed the presence of exosome markers CD9, CD63, and TSG101 in Ex, Vp.Ex, and Pg.Ex (Fig. 2c). The experimental plan for exosome tracking is presented in Fig. 2d. Confocal imaging indicated that the exosomes were initially absorbed by SCC25 cells within 6 h of incubation (Fig. 2e). These results show that *P. gingivalis*-infected OSCC cells release larger exosomes at lower concentration.

### Exosomes released from *P. gingivalis* -infected cells induce malignant phenotypes of OSCC cells

**Fig. 3:**
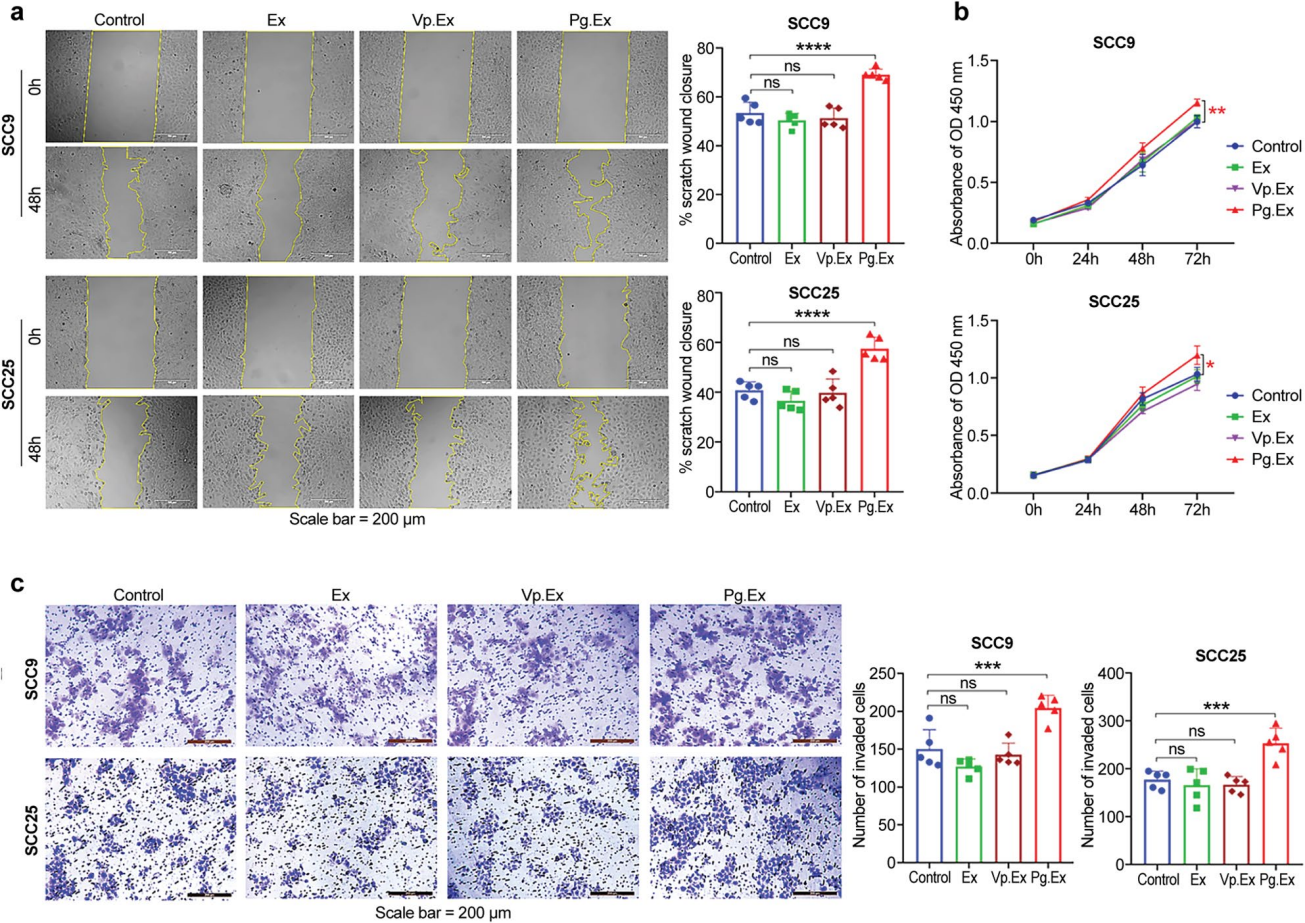
Pg.Ex promotes OSCC cell malignancy.** (a)** Scratch images of OSCC cells acquired at 0 and 48 h. Statistical analysis of the wound closure area (right). **(b)** CCK-8 assay of SCC25 and SCC9 cells incubated with Ex, Vp.Ex, or Pg.Ex. **(c)** Matrigel invasion assay of OSCC cells co-cultured with Ex, Vp.Ex, or Pg.Ex. Quantitative analysis of the Matrigel invasion assay (right). Scale bar, 200 μm. Results are presented as mean (SD). ns, not significant; *P < 0.05; **P < 0.01; ***P < 0.001; ****P < 0.0001. Data are analyzed using two-way ANOVA in **(a)** and one-way ANOVA in **(b)** and **c)**. OSCC, oral squamous cell carcinoma

We conducted a preliminary experiment to identify the optimal Pg.Ex concentration for enhancing OSCC malignancy. The pre-experiment results indicated that Pg.Ex significantly induced malignant phenotypes of OSCC cells at a concentration of 15 µg mL^− 1^ (*P* < 0.001; Additional File 2: Figure S2). SCC25 and SCC9 cells treated with Ex, Vp.Ex, or Pg.Ex showed that Pg.Ex significantly accelerated wound closure compared to Ex and Vp.Ex (both *P* < 0.0001; Fig. 3a). Subsequently, the CCK-8 assay showed that Pg.Ex enhanced the proliferation of SCC25 and SCC9 cells (*P* < 0.05, *P* < 0.01; Fig. 3b). Additionally, Transwell assays demonstrated that the invasion ability of Pg.Ex treatment was significantly increased in SCC25 and SCC9 cells compared to the Ex and Vp.Ex groups (both *P* < 0.001; Fig. 3c). These results indicate that Pg.Ex induces the malignant phenotype in non-infected OSCC cells by promoting their proliferation, migration, and invasion.

### Specific miRNAs are decreased in exosomes derived from *P. gingivalis* -infected OSCC cells

**Fig. 4:**
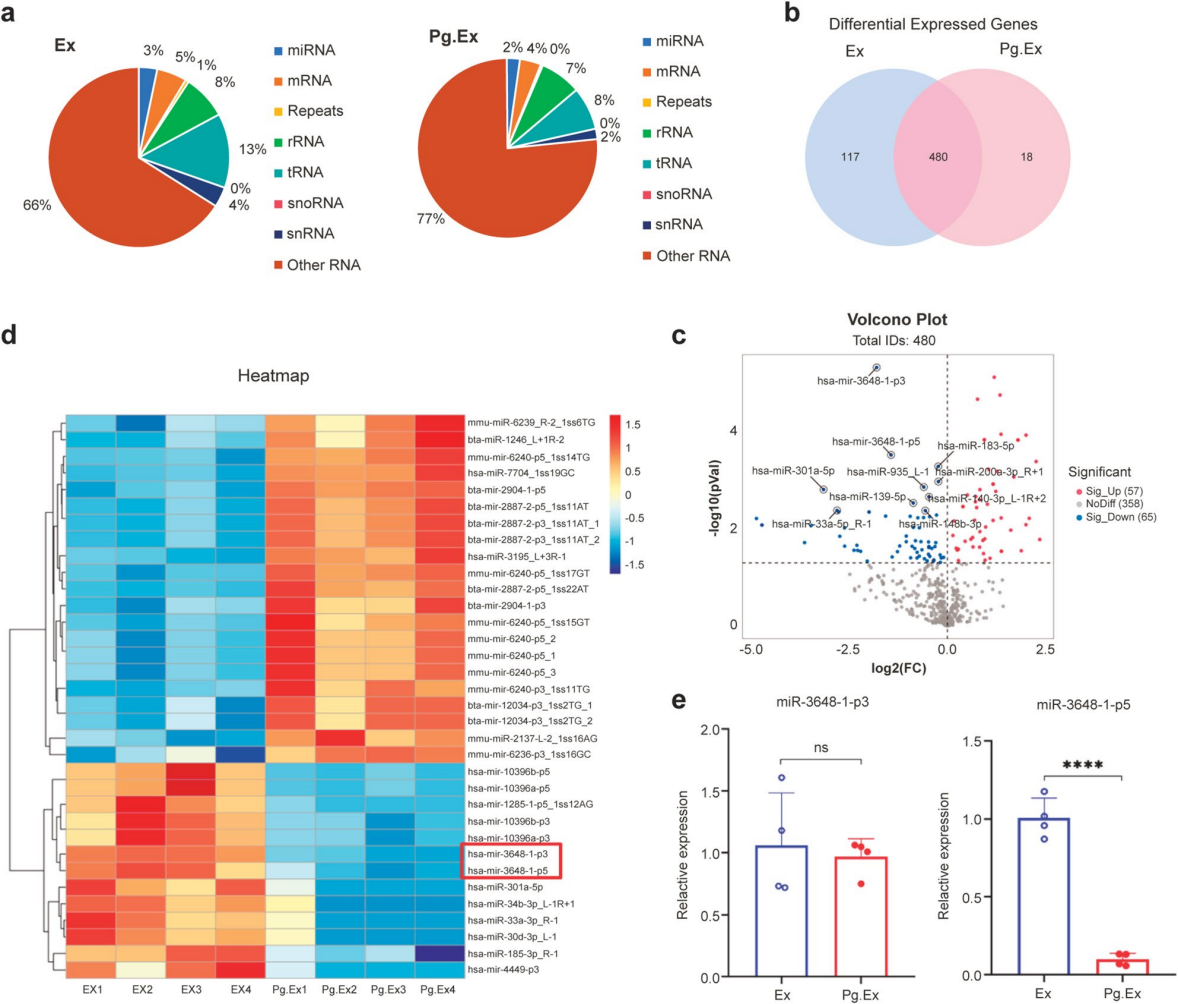
High-throughput miRNA sequencing of Ex and Pg.Ex. **(a)** Percentages of small and unlabeled RNA categories. **(b)** Venn diagram showing common and unique miRNAs between Ex and Pg.Ex. Statistical significance was set at *P <* 0.05. **(c)** Volcano plot comparing the expression levels of miRNAs in Pg.Ex and Ex (red, enhanced expression; blue, reduced expression; grey, no statistical difference). Statistical significance was set at *P <* 0.05. **(d)** Heatmap comparing the expression levels of miRNAs in Pg.Ex and Ex. *P <* 0.01; expression level = high or medium; fold change ≥ 2 or ≤ 0.5. **(e)** qRT-PCR confirms the low level of miR-3648-1-p5 in Pg.Ex. Data are presented as mean (SD) in **e)**. ns, not significant; ****P < 0.0001. Data in **e)** are analyzed using an unpaired *t*-test

To identify potential exosomal miRNAs involved in OSCC progression, we performed high-throughput miRNA sequencing to compare the differential expression profiles of Ex and Pg.Ex. MiRNAs isolated from Ex and Pg.Ex accounted for 3% and 2% of the total RNA, respectively (Fig. 4a; Table 2). A total of 597 and 498 known miRNAs were identified in both Ex and Pg.Ex, respectively (Additional File 3). A total of 480 miRNAs were expressed in both groups. The overlapping and unique numbers of miRNAs between Ex and Pg.Ex are presented in Fig. 4b and Additional File 4.

**Table 2:** Summary of the small RNA sequencing of *P. gingivalis* -infected exosomes and non-infected exosomes

Categories	Ex		Pg.Ex	
	Clean Reads	percent	Clean Reads	percent
All	29112966.25	100%	20176794.8	100%
miRNA	955456.6167	3%	462272.1917	2%
mRNA	1574723.75	5%	769313.75	4%
rRNA	2277682.5	8%	1463930.25	7%
tRNA	3819171.5	13%	1542258.5	8%
snoRNA	32534.25	0.11%	21463.25	0.11%
snRNA	1030077.25	4%	357810.25	2%
Others	19271462.88	66%	15489982.06	77%

Ex, exosomes from non-infected OSCC cells; OSCC, oral squamous cell carcinoma; *P. gingivalis*, *Porphyromonas gingivalis*; Pg.Ex, exosomes secreted from *P. gingivalis*-infected OSCC cells.

We analyzed the expression levels of overlapping miRNAs in the two groups and identified 122 miRNAs with differences (*P* < 0.05; Fig. 4c, Additional File 5). Further analysis using a heatmap revealed that 21 miRNAs were upregulated and 13 miRNAs downregulated in Pg.Ex (*P* < 0.01; expression level = high or medium; fold change ≤ 0.5 or ≥ 2) (Fig. 4d). The top five miRNAs with the lowest expression in Pg.Ex were selected for validation (*P* < 0.05; expression level = high or medium; fold change < 0.5). After excluding recently predicted unknown miRNAs, the remaining two miRNAs (miR-3648-1-p3 and miR-3648-1-p5) were validated using qRT-PCR. The decrease in miR-3648-1-p5 in Pg.Ex cells was verified by qRT-PCR (*P* < 0.0001; Fig. 4e). These results indicate that *P. gingivalis* infection regulates the exosomal miRNA profile of OSCC cells, with a reduction in miR-3648-1-p5.

### Downregulation of Exosomal miR-3648-1-p5 promotes the tumor progression of OSCC cells

**Fig. 5:**
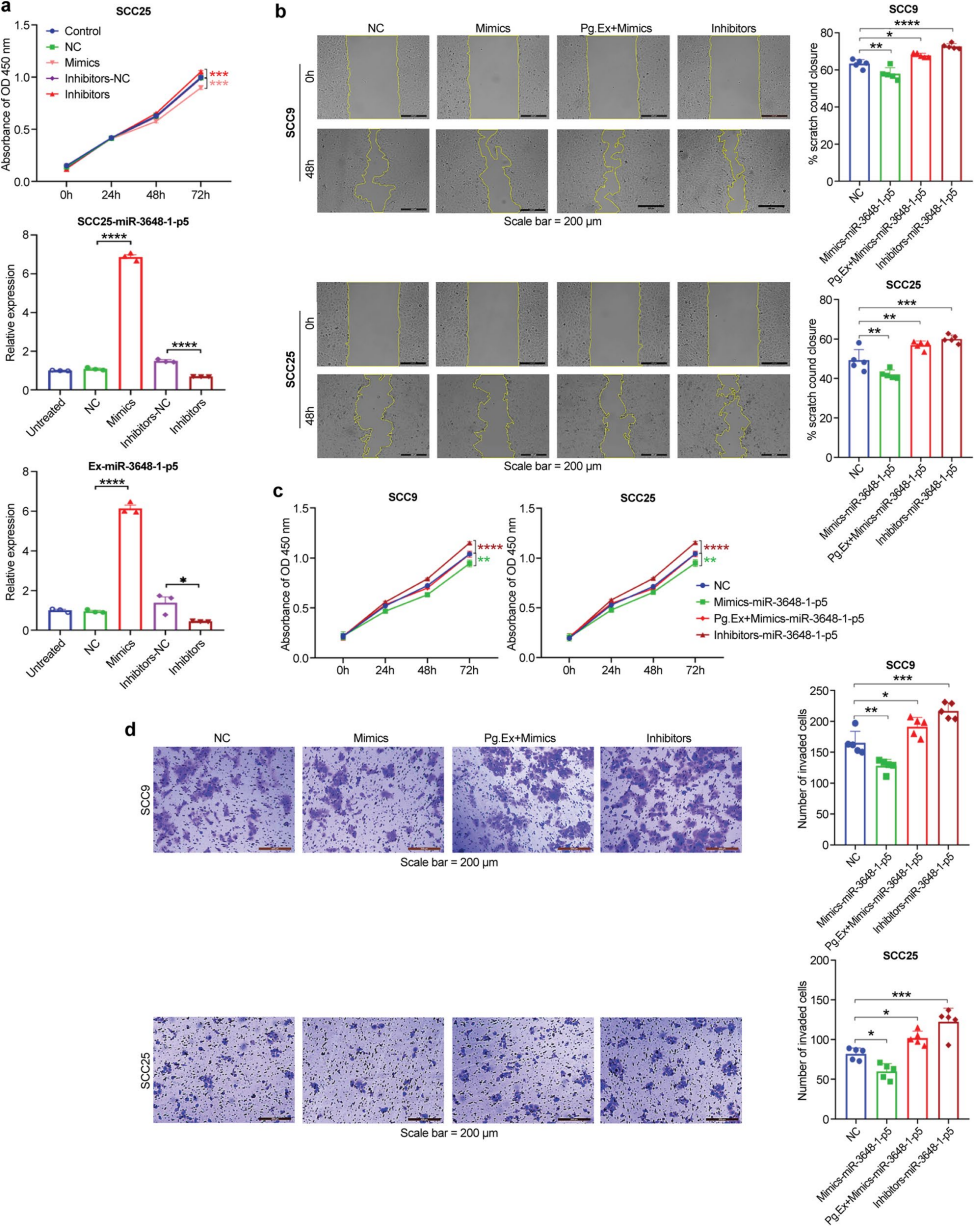
Inhibition of miR-3648-1-p5 promotes the malignancy of OSCC cells.** (a)** Transfection efficiency is evaluated using qRT-PCR for cellular and exosomal miR-3648-1-p5 (*n* = 3). **(b)** OSCC cell migration following the indicated treatments is evaluated using the scratch wound assay. **(c)** and **(d)** In addition, CCK-8 and Matrigel invasion assays are used to detect the proliferation and invasion of the cells in each group. Images are obtained at 0 and 48 h. Scale bar, 200 μm. Data are presented as mean (SD). *P < 0.05; **P < 0.01; ***P < 0.001; ****P < 0.0001. Data are analyzed using two-way ANOVA in **b)**, right of **a)**, and one-way ANOVA in **b)**, **c)**, and **d)**, left and middle of **a)**. OSCC, oral squamous cell carcinoma

To investigate the biological functions of exosomal miR-3648-1-p5 during *P. gingivalis* infection, we used miR-3648-1-p5 generated by overexpressing (mimic) and knocking down (inhibitor) miRNA (Fig. 5a). Scratch-wound assay showed that miR-3648-1-p5 inhibitors promoted the migration of SCC25 and SCC9 cells compared with the NC, while Pg.Ex partially rescued the mimic-induced low-migratory area (Fig. 5b). Compared to the NC, miR-3648-1-p5 inhibitors enhanced cell proliferation, whereas miR-3648-1-p5 mimics inhibited the proliferation of OSCC cells. The addition of Pg.Ex to the mimic-treated cells partially reversed this inhibitory effect (Fig. 5c). In addition, the Transwell invasion assay indicated that compared with the NC, miR-3648-1-p5 inhibitors greatly enhanced the invasion of SCC25 cells, whereas the combination of mimics reversed Pg.Ex-mediated invasion. A similar trend was observed in the SCC9 cells (Fig. 5d). These results indicate that Pg.Ex promotes tumor progression linked to low level exosomal miR-3648-1-p5.

### Exosomes released from *P. gingivalis*-infected OSCC cells promote tumor progression in vivo

**Fig. 6:**
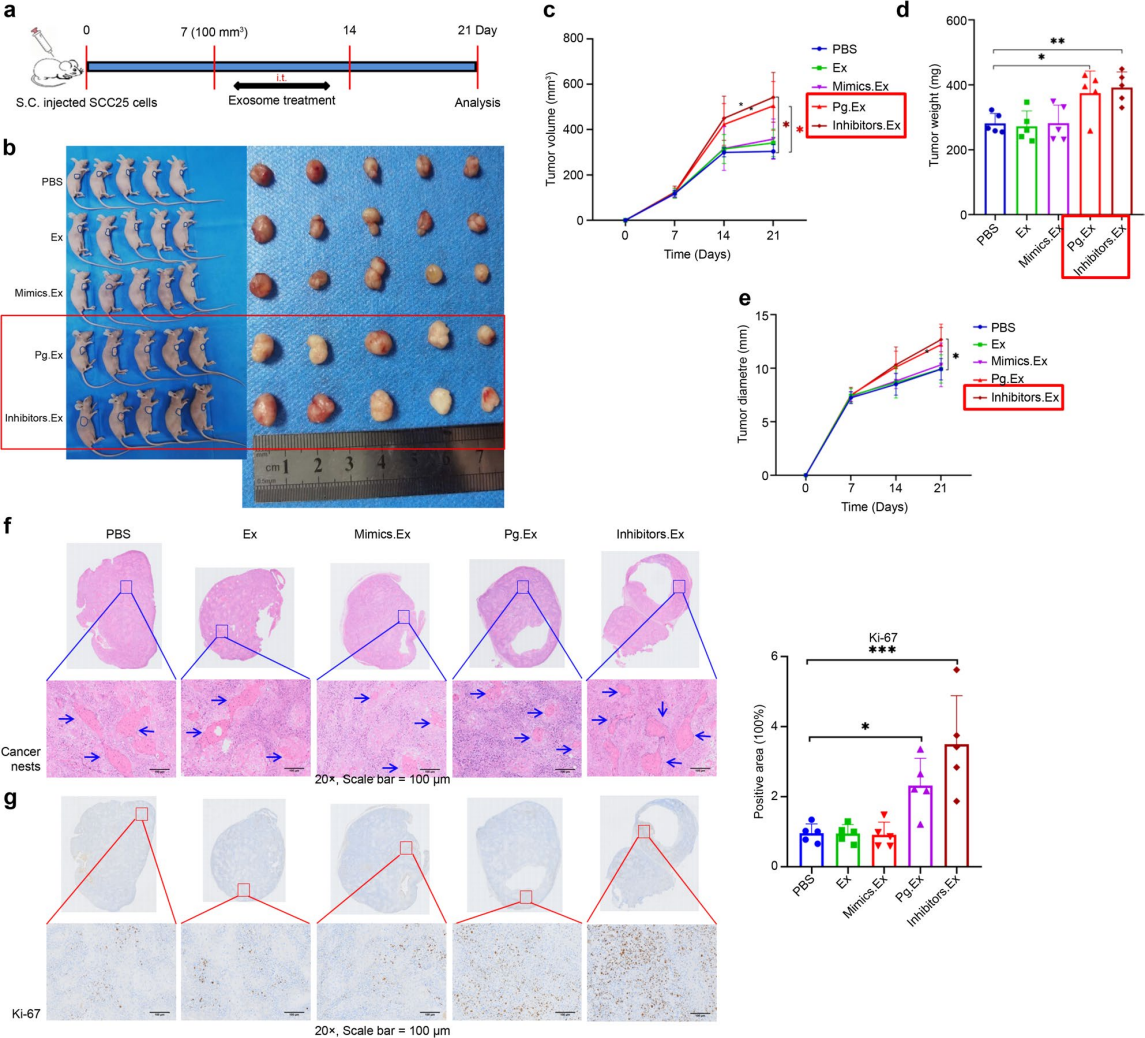
Animal experiments show that Pg.Ex and Inhibitors.Ex promote OSCC progression.** (a)** Flowchart describing the design of the in vivo experiments. **(b)** Typical images of subcutaneous tumors in nude mice treated with PBS, Ex, Mimics.Ex, Pg.Ex, or Inhibitors.Ex (*n* = 5). Quantitative analysis of xenograft tumor volume **c)**, weight **d)** and diameter **e)**. **f)** Cancer nests in tumor tissues are visualized using hematoxylin–eosin staining. Scale bar, 100 μm. **g)** Immunohistochemical staining of Ki-67 expression in tumor tissues. Scale bar, 100 μm. Data are presented as mean (SD) in **c)**, **d)**, **e)**, and **g)**. Data are analyzed using two-way ANOVA in **c)**, and **e)**, and one-way ANOVA in **d)** and **g).** *P < 0.05; **P < 0.01; ***P < 0.001. OSCC, oral squamous cell carcinoma

To assess the tumorigenic potential of *P. gingivalis*-infected exosomes and exosomal miR-3648-1-p5 in vivo, a human OSCC xenograft model was employed. First, we conducted a preliminary animal experiment to determine the most effective concentration of Pg.Ex to promote OSCC progression. The results indicated that Pg.Ex significantly increased tumor volume, weight, and diameter at a concentration of 10 µg per time (*P*-values are 0.0342, 0.0151 and 0.0107, respectively; Additional File 2: Figure S3). PBS was used as the control. The procedure for the final animal experiment was the same as that for the preliminary animal experiment (Fig. 6a) [20]. Pg.Ex and Inhibitors.Ex enhanced tumor growth compared with PBS (Fig. 6b). Tumors in the Inhibitors.Ex group were larger (*P* < 0.05), heavier (*P* < 0.01), and had a longer diameter (*P* < 0.05), along with higher expression levels of the proliferation marker Ki-67 (*P* < 0.001), compared to the PBS group (*P* < 0.05; Fig. 6c–e, g). A similar trend was observed in the Pg.Ex group (*P* < 0.05; Fig. 6c, d, g). No significant differences in body weight were observed among the groups (Additional file 2: Figure S4). In addition, hematoxylin–eosin staining revealed the presence of cancer nests in all groups (Fig. 6f). These results indicate that exosomes from *P. gingivalis*-infected OSCC cells promote tumor progression by carrying a low level of exosomal miR-3648-1-p5.

## Discussion

Recent advances in immunotherapy, targeted therapy, and anti-angiogenic treatments have improved OSCC outcomes; however, its global incidence continues to increase [25]. Therefore, identifying pathogenic factors and exploring more effective treatment strategies to improve patient prognosis is crucial. Several studies have reported a strong association between chronic inflammation induced by bacterial infection and tumor metastasis [4]. It is well established that *P. gingivalis* is an important pathogenic factor in OSCC growth, consistent with our previous research [6]. Patients infected with *P. gingivalis* are more likely to develop aggressive OSCC compared to non-infected patients.

Interestingly, in our study, we found that exosomes from OSCC cells carrying *P. gingivalis* played a crucial role in promoting OSCC progression. Furthermore, we observed that *P. gingivalis*-infected OSCC cells released larger and fewer exosomes, a finding that differs from the exosome characteristics observed in other bacteria such as *F. nucleatum* ^20^, *Mycobacteria* [28], and *Legionella pneumophila* [29]. Previous studies have shown that exosome concentration and content are influenced by factors such as cell source, cell type, intervention conditions, and cell state [30]. The formation of outer membrane vesicles from *P. gingivalis* may largely depend on the expression of *fimA* [31]. *P. gingivalis* strains are classified into six types (type I and Ib–V) based on the genotype of the main subunit FimA in the pili. The type IV (W83) strain secreted fewer outer membrane vesicles than the type I (ATCC 33277) and type III (ATCC 49417) strains. Similarly, *P. gingivalis* W83 infection may cause a decrease in the secretion of exosomes in host OSCC cells. This may explain the differences in exosome characteristics compared to other bacteria such as *F. nucleatum*^20^, *Mycobacteria* [28], and *L. pneumophila* [29].

Accumulating evidence has shown that exosomes released from host cells carrying pathogens can mediate intercellular communication and information transmission by transporting substances with host cell biological characteristics such as lipids, proteins, RNA, and DNA [32]. However, intercellular communication between *P. gingivalis*-infected OSCC cells and non-infected OSCC cells remains poorly understood. Lötvall [33] first discovered the transmission of miRNAs through exosomes and proposed that this mechanism could serve as a major mode of intercellular genetic exchange. This discovery has sparked a strong interest in investigating the functions of exosomal miRNAs secreted by *P. gingivaliss*-infected OSCC cells in OSCC progression.

Typically, bacteria modulate host miRNAs to facilitate bacterial survival and replication [20, 28, 29]. Similarly, *P. gingivalis* may regulate miRNAs in infected OSCC cells, potentially contributing to the survival of *P. gingivalis* in cancerous tissues. In this study, our findings have confirmed this hypothesis both in vitro and in vivo and revealed that the number of exosomes and exosomal miR-3648-1-p5 derived from *P. gingivalis*-infected OSCC cells was reduced. These exosomes could be subsequently endocytosed by neighboring OSCC cells not infected by *P. gingivalis*, promoting tumor progression. However, the biological functions and mechanisms of exosomal miR-3648-1-p5 remain largely unexplored in cancer. To investigate the potential biological functions of exosomal miR-3648-1-p5 during *P. gingivalis* infection, we conducted in vitro and in vivo experiments using miR-3648-1-p5 mimics and inhibitors. Our findings revealed that Pg.Ex promotes malignant phenotypes in OSCC, linked to low levels of exosomal miR-3648-1-p5. *P. gingivalis* infection reduced the secretion of Pg.Ex and led to lower levels of exosomal miR-3648-1-p5, thereby enhancing carcinogenic effects.

Our study has some limitations. First, our findings demonstrated that miR-3648-1-p5 mimics can inhibit the malignant phenotype of OSCC cells in vitro. However, Mimics.Ex did not show tumor inhibitory effects in our animal experiments. We believe this may be attributed to two factors: (1) the intervention time of exosomes was too short to allow Mimics.Ex to inhibit OSCC progression; and (2) given the scarcity of research related to miR-3648-1-p5 and the complex regulatory network in animals, there may be other factors in vivo that can regulate miR-3648-1-p5. The molecular mechanism underlying the miR-3648-1-p5 in OSCC remains unexplored. Future experiments should include bioinformatics analyses to predict and evaluate miR-3648-1-p5 targets using algorithms such as TargetScan and miRanda. This will help investigate the underlying molecular mechanisms and clinical applications of exosomal miR-3648-1-p5 secreted by *P. gingivalis* -infected OSCC cells. Second, our preliminary animal results indicated that Pg.Ex had a negative effect on lymph node (LN) metastasis in oral cancer. It has been reported that the risk of nodal metastasis depends on multiple complicated factors. This may be related to three factors: (1) Pg.Ex only promotes the progression of primary OSCC tumors and cannot promote LN metastasis of oral cancer; (2) the intervention time of exosomes was too short to promote LN metastasis in oral cancer; and (3) primary tumors were small and at a volume that was not sufficient to promote LN metastasis. LN metastasis is positively correlated with the volume of the primary tumor [34, 35]. As lymphangiogenesis is the rate-limiting step for LN metastasis in esophageal squamous cell carcinoma [35], future studies should investigate the effects of Pg.Ex and exosomal miR-3648-1-p5 on tube formation and the migration of human lymphatic endothelial cells.

This study presents three key innovations. First, we performed high-throughput miRNA sequencing on Pg.Ex and Ex to investigate the differential miRNA expression profiles between Pg.Ex and Ex. Our analysis revealed 57 upregulated and 65 downregulated miRNAs in Pg.Ex compared with Ex. Second, we found that exosome production and secretion from OSCC cells are regulated by *P. gingivalis* infection. Pg.Ex was larger and had a lower concentration than Ex and Vp.Ex. Finally, we discovered that low expression levels of exosomal miR-3648-1-p5 isolated from *P. gingivalis*-infected OSCC cells were involved in OSCC progression.

## Conclusion

The present study found that *P. gingivalis* infection regulates exosome production and secretion from OSCC cells. Pg.Ex was larger in size and lower in concentration than Ex and Vp.Ex. Our results reveal the mechanism by which low levels of exosomal miR-3648-1-p5 secreted by *P. gingivalis*-infected OSCC cells are strongly associated with OSCC progression. Our research provides insights into the molecular mechanism by which *P. gingivalis* contributes to the development of OSCC. By enhancing our understanding of the relationship between *P. gingivalis* and OSCC, a prevention and treatment strategy for OSCC patients with *P. gingivalis* infection may be developed.

## Supplementary Information

Below is the link to the electronic supplementary material.

Supplementary Material 1

Supplementary Material 2

Supplementary Material 3

Supplementary Material 4

Supplementary Material 5

## Data Availability

The datasets generated during and/or analyzed during the current study are available from the corresponding author on reasonable request.

## Abbreviations

Abbreviation: Full term
CFUs: Colony-forming units
EVs: Extracellular vesicles
Ex: Exosomes from non-infected OSCC cells
FBS: Fetal bovine serum
F. nucleatum: Fusobacterium nucleatum
H&E: Hematoxylin-eosin
IHC: Immunohistochemistry
Inhibitors.Ex: Exosomes secreted by OSCC cells transfected with miR-3648-1-p5 inhibitors
Mimics.Ex: Exosomes secreted by OSCC cells transfected with miR-3648-1-p5 mimics
MOI: Multiplicity of infection
NC: Negative control
NTA: Nanoparticle Tracking Analyzers
PBS: Phosphate buffered saline
OSCC: Oral squamous cell carcinoma
P. gingivalis: Porphyromonas gingivalis
Pg.Ex: Exosomes from P. gingivalis -infected OSCC cells
V. parvula: Veillonella parvula
Vp.Ex: Exosomes from V. parvula -infected OSCC cells
