# Supplementary material for: Association of Porphyromonas gingivalis-infected oral squamous cell carcinoma cell-secreted exosomal miR-3648-1-p5 with tumor progression
Source: Cancer Cell Int. 2026 Mar 4;26:163. doi: 10.1186/s12935-026-04230-5 (PMC13069696; doi:10.1186/s12935-026-04230-5)
Supplement: Supplementary file 1 — Supplementary Material 1 [file 12935_2026_4230_MOESM1_ESM.docx]

**Additional File 1. Manuscripts reporting high-throughput screens of small-molecule libraries**

**The methods and procedures used to conduct the Small RNA experimental**

The RNA concentration and purity of each sample were evaluated using the NanoDrop ND-1000 (NanoDrop, Wilmington, DE, USA). RNA fragment integrity was evaluated using the Bioanalyzer 2100 (Agilent, CA, USA). The obtained total RNA were denaturated at 70°C for 2 min with DNA 3’ Adapters, and then mixed with T4 RNA Ligase 2, truncated K227Q (NEB, M0351L, USA) at room temperature. The 3’ adapter ligation reaction was conducted at 16°C for more than 8 hours. The rest of the 3’Adapters were removed using RTP at 37°C for 30 min. The 5’ adapters and the products from the last step were then ligated with T4 RNA Ligase 1 (NEB, M0204L, USA) at 37°C for 60 min, after which a reverse-transcription reaction was carried out using SuperScript II Reverse Transcriptase (Thermo, 18064014, USA) at 50°C for 60 min and 80°C for 10 min. The cDNA products were amplified using Phusion® High-Fidelity DNA Polymerase (NEB, M0530L, USA), according to the following cycling protocol: denaturation step at 98°C for 30 sec, followed by an annealing step at 60°C for 30 sec, and an extension step at 72°C for 15 sec, repeated for 10–16 cycles. The final 5-minute extension was conducted at 72°C. The PCR products were purified and enriched through PAGE electrophoresis. The small RNA sequencing library was prepared using the TruSeq Small RNA Sample Prep kits (Illumina, San Diego, USA) kit. Following preparation of the library, the sequencing strategy was Single-end 50 bp for Illumina Hiseq 2500 following the vendor’s recommended protocol. Data analysis was conducted using the miRNA data analysis software ACGT101 miR (LC Sciences, Houston, Texas, USA) independently developed by Lianchuan Biotechnology.

**The bioinformatics analysis methods of miRNA**

Raw reads were subjected to an in-house program, ACGT101-miR (LC Sciences, Houston, Texas, USA) to remove adapter dimers, junk, low complexity, common RNA families (rRNA, tRNA, snRNA, snoRNA), and repeats. Subsequently, unique sequences with length in 18-26 nucleotides were mapped to specific species precursors in miRBase 22.0 using BLAST search to identify known miRNAs and novel 3p- and 5p- derived miRNAs. Length variation at both the 3’ and 5’ ends and one mismatch inside of the sequence were allowed in the alignment. The unique sequences which mapped hairpin arms of mature miRNAs of specific species were identified as known miRNAs. Unique sequences mapping to the other arm of known specific species precursor hairpins opposite to the annotated mature miRNA-containing arm were considered to be novel 5p- or 3pderived miRNA candidates. The remaining sequences were mapped to other selected species precursors (with the exclusion of specific species) in miRBase 22.0 using BLAST search, and the mapped pre-miRNAs were further BLASTed against the specific species genomes to determine their genomic locations. The above two types were defined as known miRNAs. Unmapped sequences were BLASTed against the specific genomes, and the hairpin RNA structures containing sequences were predicated from the flank 80 nt sequences using RNAfold software (http://rna.tbi.univie.ac. at/cgi-bin/RNAfold.cgi). The criteria for secondary structure prediction were as follows: (1) number of nucleotides in one bulge in stem ≤12; (2) number of base pairs in the stem region of the predicted hairpin ≥16; (3) cutoff of free energy ≤-15 kCal/mol; (4) length of hairpin (up and down stems + terminal loop) ≥50 (5) length of hairpin loop ≤20; (6) number of nucleotides in one bulge in mature region ≤8; (7) number of biased errors in one bulge in mature region ≤4; (8) number of biased bulges in the mature region ≤2; (9) number of errors in the mature region ≤7; (10) number of base pairs in the mature region of the predicted hairpin ≥12; and (11) percent of mature in stem ≥80%.

**Analysis of differentially expressed miRNAs**

Differential expression of miRNAs based on normalized deep-sequencing counts was analyzed by selectively using Fisherexact test, Chi-squared 2X2 test, Chi-squared nXn test, Student t test, or ANOVA, depending on the experimental design. The significance threshold was set at either 0.01 and 0.05 in each test.
