# Supplementary material for: Association of Porphyromonas gingivalis-infected oral squamous cell carcinoma cell-secreted exosomal miR-3648-1-p5 with tumor progression
Source: Cancer Cell Int. 2026 Mar 4;26:163. doi: 10.1186/s12935-026-04230-5 (PMC13069696; doi:10.1186/s12935-026-04230-5)
Supplement: Supplementary file 2 — Supplementary Material 2 [file 12935_2026_4230_MOESM2_ESM.docx]

**Additional File 2. Supplementary Figures and tables**

**
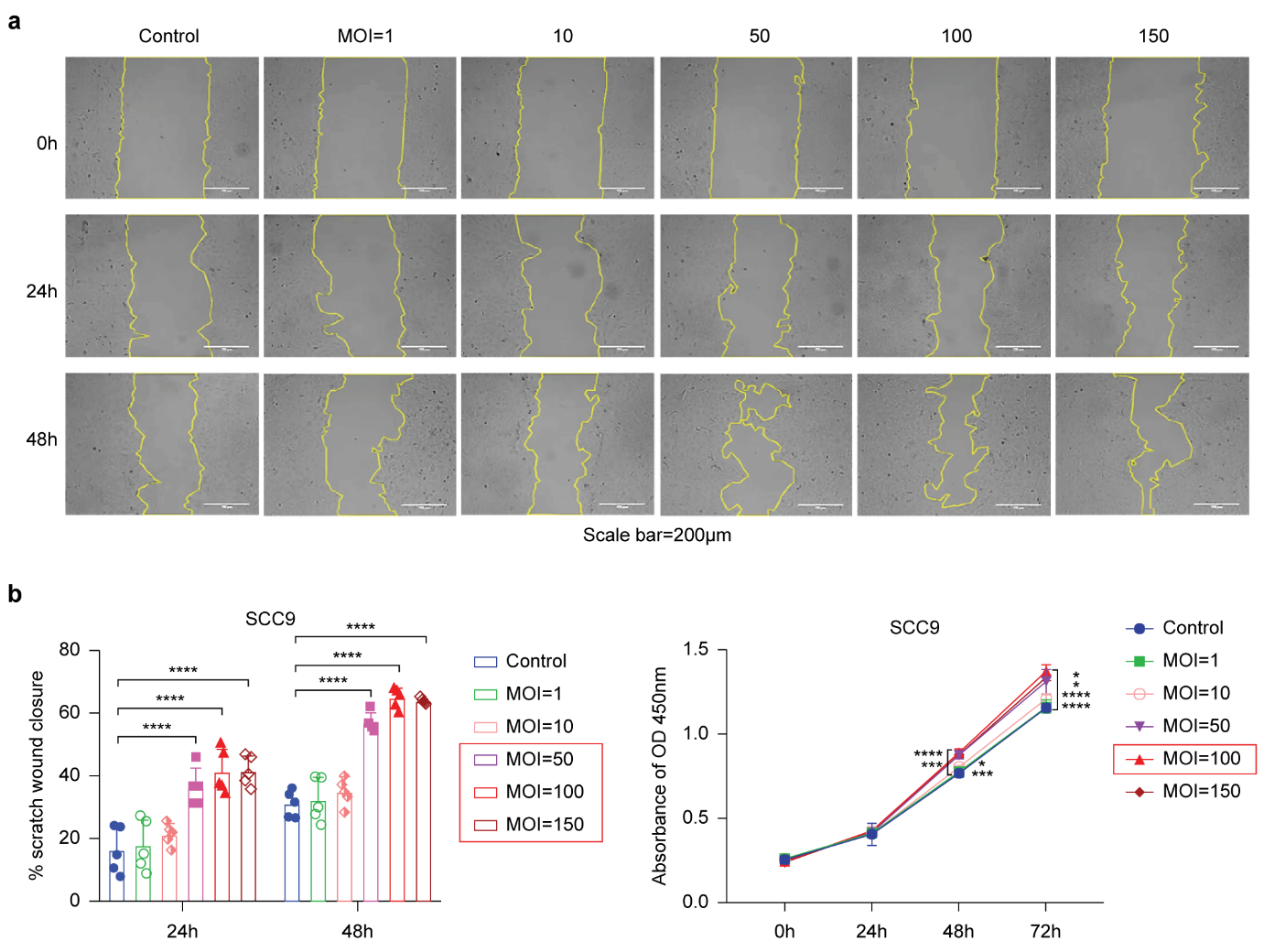
**

**Figure S1.** Co-culture duration for all experiments

**
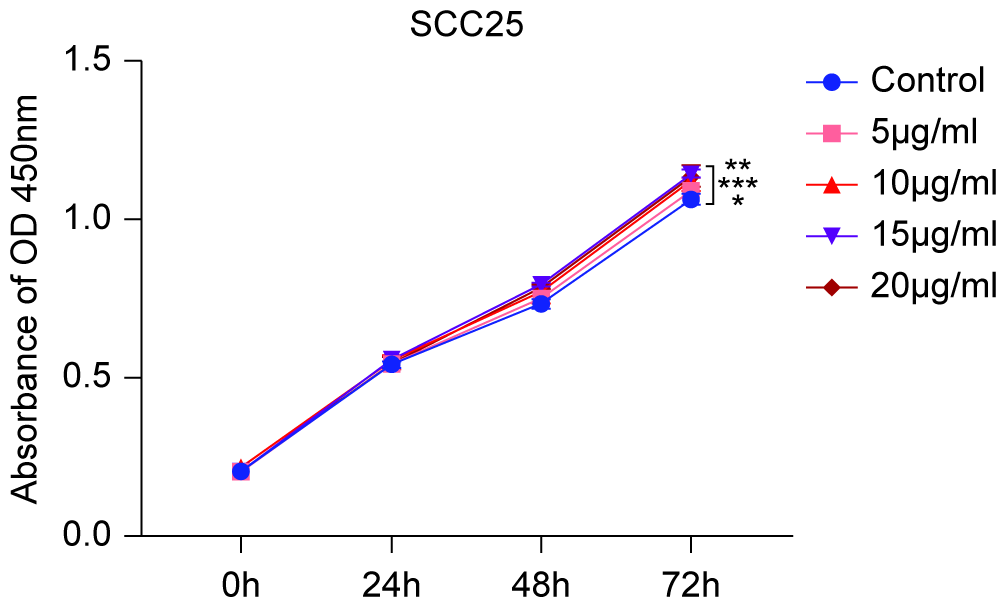
**

**Figure S2.** CCK-8 assay was used to detect the proliferation ability of SCC25 cells at different intervention concentrations of exsomes (0, 5, 10, 15, and 20 μg/mL).

**
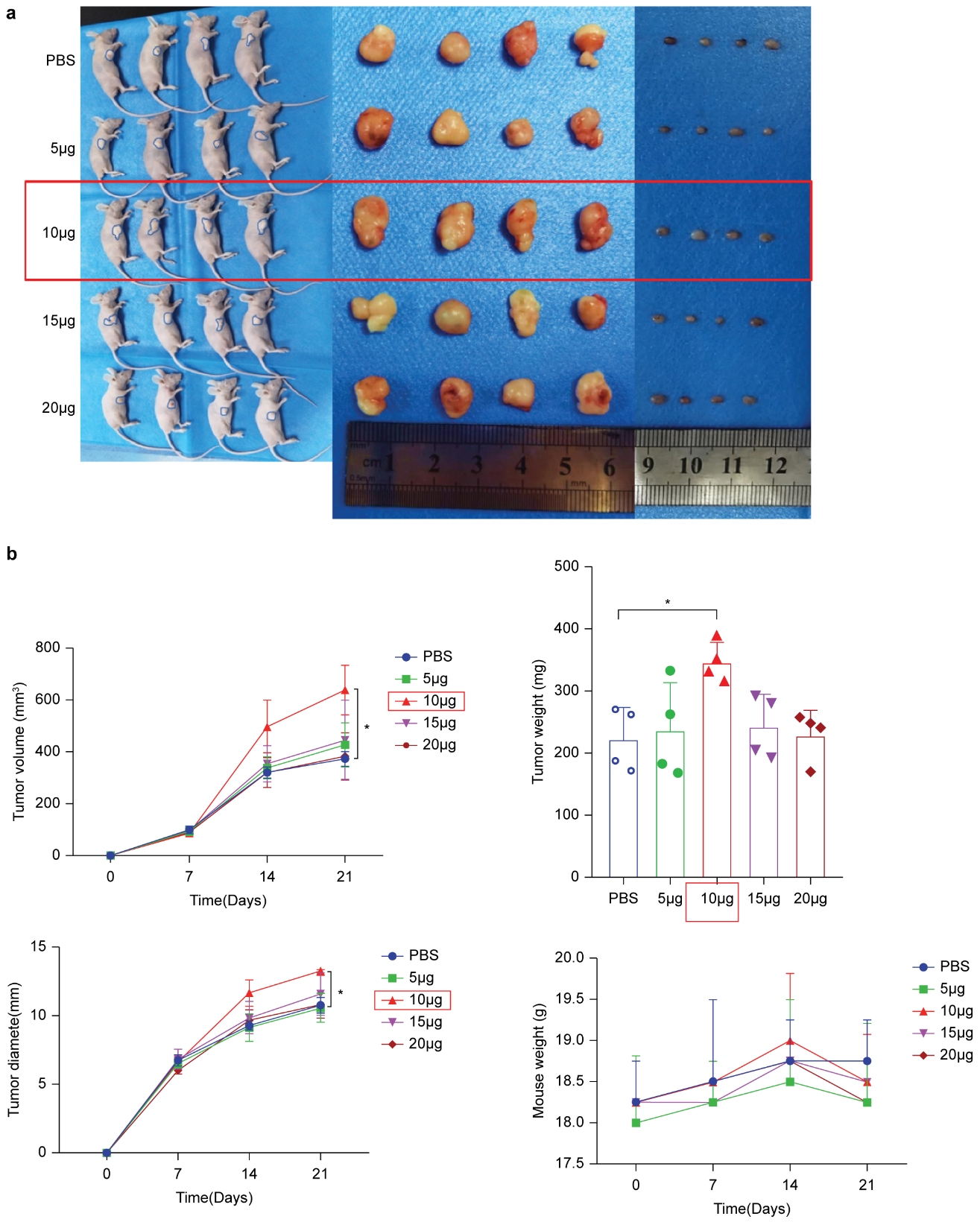
**

**Figure S3.** Preliminary animal experiments showed that Pg.Ex promoted OSCC progression at a concentration of 10ug /mice /per injection.


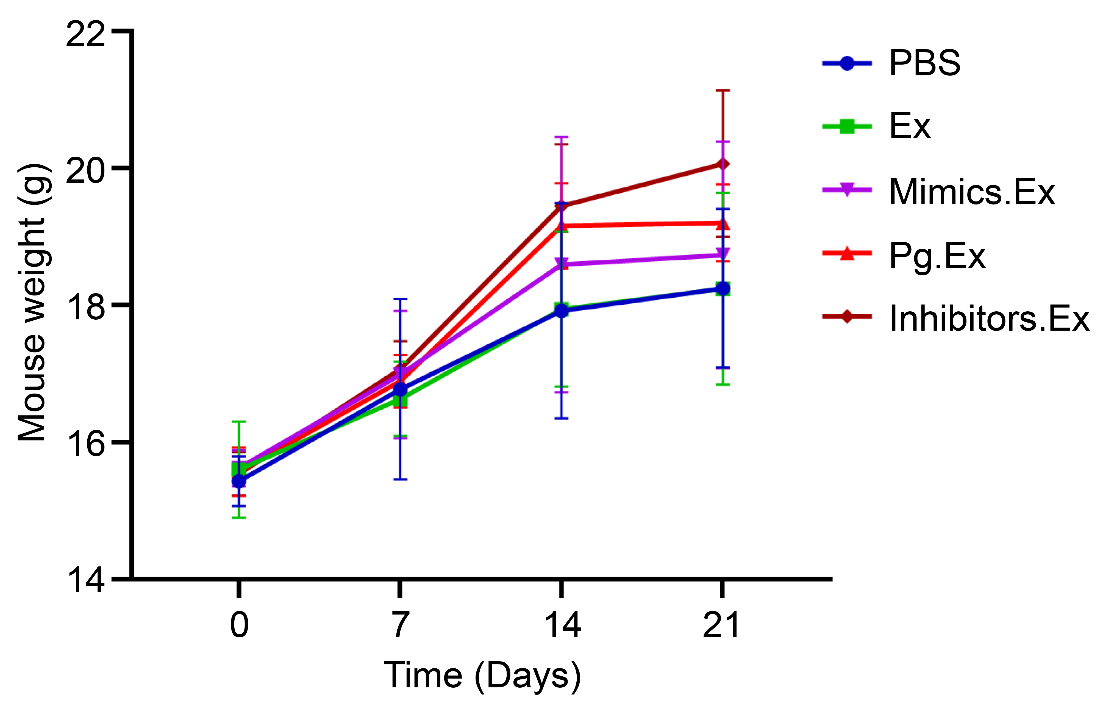


**Figure S4.** Quantitative analysis of mouse weight

**Table S1.** Primer sequences

| **miRNA** | **Primer (5′→3′)** |
| --- | --- |
| miR-3648-1-p3 | GCTCGAGGGGTCCCCGTG |
| miR-3648-1-p5 | ATCGCCGAGGGCCGGTCG |
| U6 Fw | GGAACGATACAGAGAAGATTAGC |
| U6 Rv | TGGAACGCTTCACGAATTTGCG |

Fw, forward; miRNA, microRNA; Rv, reverse
