# Supplementary material for: Association of Porphyromonas gingivalis-infected oral squamous cell carcinoma cell-secreted exosomal miR-3648-1-p5 with tumor progression
Source: Cancer Cell Int. 2026 Mar 4;26:163. doi: 10.1186/s12935-026-04230-5 (PMC13069696; doi:10.1186/s12935-026-04230-5)
Supplement: Supplementary file 3 — Supplementary Material 3 [file 12935_2026_4230_MOESM3_ESM.docx]

**Additional File 3.** Number of miRNAs identified in Ex and *Pg*.Ex

Ex: Exosomes secreted from non-infected OSCC cells

*Pg*.Ex: Exosomes secreted from *Pg*-infected OSCC cells

| Ex | Pg.Ex |
| --- | --- |
| hsa-miR-1-3p | hsa-let-7f-5p |
| hsa-let-7f-5p | hsa-let-7f-1-3p_1ss22CT |
| hsa-let-7f-1-3p_1ss22CT | hsa-let-7d-5p |
| hsa-let-7d-5p | hsa-let-7d-3p |
| hsa-let-7d-3p | hsa-let-7e-5p |
| hsa-let-7e-5p | hsa-miR-7-5p |
| hsa-let-7e-3p_R-1 | hsa-let-7a-5p |
| hsa-miR-7-5p | hsa-let-7a-3p |
| hsa-let-7a-5p | hsa-let-7i-5p |
| hsa-let-7a-3p | hsa-let-7i-3p |
| hsa-let-7i-5p | hsa-let-7c-5p |
| hsa-let-7i-3p | hsa-let-7g-5p |
| hsa-let-7c-5p | hsa-let-7g-3p_R+1 |
| hsa-let-7g-5p | hsa-let-7b-5p |
| hsa-let-7g-3p_R+1 | hsa-let-7b-3p_1ss22CT |
| hsa-let-7b-5p | hsa-miR-7-1-3p |
| hsa-let-7b-3p_1ss22CT | hsa-let-7a-3p_R+1 |
| hsa-miR-7-1-3p | hsa-let-7f-2-3p_1ss22CT |
| hsa-let-7a-3p_R+1 | hsa-miR-9-5p |
| hsa-let-7f-2-3p_1ss22CT | hsa-miR-10a-5p_R-1 |
| hsa-let-7a-2-3p | hsa-miR-10a-3p_R-1 |
| hsa-miR-9-5p | hsa-miR-10b-5p_R-1 |
| hsa-miR-9-3p | hsa-miR-15a-5p_R-1 |
| hsa-miR-10a-5p_R-1 | hsa-miR-15b-5p |
| hsa-miR-10a-3p_R-1 | hsa-miR-15b-3p_R-1 |
| hsa-miR-10b-5p_R-1 | hsa-miR-16-5p |
| hsa-miR-15a-5p_R-1 | hsa-miR-16-1-3p |
| hsa-miR-15a-3p | hsa-miR-16-2-3p_L+1R-1 |
| hsa-miR-15b-5p | hsa-miR-17-5p |
| hsa-miR-15b-3p_R-1 | hsa-miR-17-3p |
| hsa-miR-16-5p | hsa-miR-18a-5p |
| hsa-miR-16-1-3p | hsa-miR-18a-3p |
| hsa-miR-16-2-3p_L+1R-1 | hsa-miR-19b-3p |
| hsa-miR-17-5p | hsa-miR-19a-3p |
| hsa-miR-17-3p | hsa-miR-20a-5p |
| hsa-miR-18a-5p | hsa-miR-20a-3p_L-1R+1 |
| hsa-miR-18a-3p | hsa-miR-21-5p_R+1 |
| hsa-miR-19b-3p | hsa-miR-21-3p |
| hsa-miR-19b-1-5p | hsa-miR-22-5p |
| hsa-miR-19a-3p | hsa-miR-22-3p |
| hsa-miR-20a-5p | hsa-miR-23a-5p_R-1 |
| hsa-miR-20a-3p_L-1R+1 | hsa-miR-23a-3p_R+1 |
| hsa-miR-21-5p_R+1 | hsa-miR-23b-3p_R-1 |
| hsa-miR-21-3p | hsa-miR-24-3p_R-2 |
| hsa-miR-22-5p | hsa-miR-24-2-5p_L+1R-1 |
| hsa-miR-22-3p | hsa-miR-25-3p |
| hsa-miR-23c_1ss14TG | hsa-miR-26a-5p |
| hsa-miR-23a-3p_R+1 | hsa-miR-26b-5p_R+1 |
| hsa-miR-23b-5p | hsa-miR-27a-5p |
| hsa-miR-23b-3p_R-1 | hsa-miR-27a-3p_R-1 |
| hsa-miR-24-1-5p_L+1 | hsa-miR-27b-5p |
| hsa-miR-24-3p_R-2 | hsa-miR-27b-3p |
| hsa-miR-24-2-5p_L+1R-1 | hsa-miR-28-5p |
| hsa-miR-25-5p_R+2 | hsa-miR-28-3p |
| hsa-miR-25-3p | hsa-miR-29a-3p |
| hsa-miR-26a-5p | hsa-miR-29b-3p |
| hsa-miR-26b-5p_R+1 | hsa-miR-29c-3p |
| hsa-miR-27a-5p | hsa-miR-30a-5p_R+2 |
| hsa-miR-27a-3p_R-1 | hsa-miR-30a-3p |
| hsa-miR-27b-5p | hsa-miR-30c-5p_R+1 |
| hsa-miR-27b-3p | hsa-miR-30c-2-3p |
| hsa-miR-28-5p | hsa-miR-30b-5p |
| hsa-miR-28-3p | hsa-miR-30d-5p_R+2 |
| hsa-miR-29a-3p | hsa-miR-30d-3p_L-1 |
| hsa-miR-29b-3p | hsa-miR-30e-5p_R+2 |
| hsa-miR-29c-3p | hsa-miR-30e-3p_1ss22CT |
| hsa-miR-30a-5p_R+2 | hsa-miR-31-5p_R+1 |
| hsa-miR-30a-3p | hsa-miR-31-3p_R+1 |
| hsa-miR-30c-5p_R+1 | hsa-miR-32-5p |
| hsa-miR-30c-1-3p_R-2 | hsa-miR-32-3p_R-1 |
| hsa-miR-30c-2-3p | hsa-miR-33a-3p_R-1 |
| hsa-miR-30b-5p | hsa-miR-34a-5p |
| hsa-miR-30d-5p_R+2 | hsa-miR-34b-5p_L-1R+1 |
| hsa-miR-30d-3p_L-1 | hsa-miR-34b-3p_L-1R+1 |
| hsa-miR-30e-5p_R+2 | hsa-miR-34c-5p |
| hsa-miR-30e-3p_1ss22CT | hsa-miR-34c-3p |
| hsa-miR-31-5p_R+1 | hsa-miR-92b-3p |
| hsa-miR-31-3p_R+1 | hsa-miR-92a-3p |
| hsa-miR-32-5p | hsa-miR-93-5p |
| hsa-miR-32-3p_R-1 | hsa-miR-93-3p_R+1 |
| hsa-miR-33a-5p_R-1 | hsa-miR-95-3p_R-1 |
| hsa-miR-33a-3p_R-1 | hsa-miR-96-5p |
| hsa-miR-34a-5p | hsa-miR-98-5p |
| hsa-miR-34b-5p_L-1R+1 | hsa-miR-98-3p_1ss22CT |
| hsa-miR-34b-3p_L-1R+1 | hsa-miR-99a-5p_R-1 |
| hsa-miR-34c-5p | hsa-miR-99b-5p |
| hsa-miR-34c-3p | hsa-miR-99b-3p_R+1 |
| hsa-miR-92b-5p_R+2 | hsa-miR-100-5p |
| hsa-miR-92b-3p | hsa-miR-101-3p_R+1 |
| hsa-miR-92a-3p | hsa-miR-103a-2-5p |
| hsa-miR-93-5p | hsa-miR-103a-3p |
| hsa-miR-93-3p_R+1 | hsa-miR-106b-5p |
| hsa-miR-95-3p_R-1 | hsa-miR-106b-3p |
| hsa-miR-96-5p | hsa-miR-106a-5p_1ss1AC |
| hsa-miR-98-5p | hsa-miR-107_R-2 |
| hsa-miR-98-3p_1ss22CT | hsa-miR-122-5p_R-1 |
| hsa-miR-99a-5p_R-1 | hsa-miR-125b-5p |
| hsa-miR-99b-5p | hsa-miR-125a-5p_R-1 |
| hsa-miR-99b-3p_R+1 | hsa-miR-125a-3p_R-1 |
| hsa-miR-100-5p | hsa-miR-125b-1-3p_R-1 |
| hsa-miR-101-3p_R+1 | hsa-miR-126-5p |
| hsa-miR-103a-2-5p | hsa-miR-126-3p_R-1 |
| hsa-miR-103a-3p | hsa-miR-127-3p |
| hsa-miR-106b-5p | hsa-miR-128-3p |
| hsa-miR-106b-3p | hsa-miR-129-5p |
| hsa-miR-106a-5p_1ss1AC | hsa-miR-129-1-3p |
| hsa-miR-107_R-2 | hsa-miR-130b-5p_R+1 |
| hsa-miR-122-5p_R-1 | hsa-miR-130b-3p |
| hsa-miR-125b-5p | hsa-miR-130a-5p |
| hsa-miR-125a-5p_R-1 | hsa-miR-130a-3p |
| hsa-miR-125a-3p_R-1 | hsa-miR-132-5p |
| hsa-miR-125b-1-3p_R-1 | hsa-miR-132-3p |
| hsa-miR-126-5p | hsa-miR-134-5p |
| hsa-miR-126-3p_R-1 | hsa-miR-135b-5p |
| hsa-miR-127-5p | hsa-miR-135b-3p |
| hsa-miR-127-3p | hsa-miR-136-3p |
| hsa-miR-128-3p | hsa-miR-138-5p |
| hsa-miR-129-5p | hsa-miR-139-5p |
| hsa-miR-129-1-3p | hsa-miR-140-5p |
| hsa-miR-130b-5p_R+1 | hsa-miR-140-3p_L-1R+2 |
| hsa-miR-130b-3p | hsa-miR-141-3p_R+1 |
| hsa-miR-130a-5p | hsa-miR-143-3p_R+1 |
| hsa-miR-130a-3p | hsa-miR-146a-5p |
| hsa-miR-132-5p | hsa-miR-146b-5p_R+1 |
| hsa-miR-132-3p | hsa-miR-148b-5p_L+1 |
| hsa-miR-134-5p | hsa-miR-148b-3p |
| hsa-miR-135b-5p | hsa-miR-148a-5p |
| hsa-miR-136-3p | hsa-miR-148a-3p |
| hsa-miR-138-5p | hsa-miR-149-5p_R-3 |
| hsa-miR-139-5p | hsa-miR-151b_R+2 |
| hsa-miR-139-3p | hsa-miR-151a-5p |
| hsa-miR-140-5p | hsa-miR-151a-3p |
| hsa-miR-140-3p_L-1R+2 | hsa-miR-152-3p |
| hsa-miR-141-3p_R+1 | hsa-miR-154-3p |
| hsa-miR-143-3p_R+1 | hsa-miR-155-5p_R-1 |
| hsa-miR-146a-5p | hsa-miR-181a-5p |
| hsa-miR-146b-5p_R+1 | hsa-miR-181a-2-3p |
| hsa-miR-148b-5p_L+1 | hsa-miR-181b-5p_R+1 |
| hsa-miR-148b-3p | hsa-miR-181c-5p_R+2 |
| hsa-miR-148a-5p | hsa-miR-181c-3p_L-1R+1 |
| hsa-miR-148a-3p | hsa-miR-181d-5p_R+1 |
| hsa-miR-149-5p_R-3 | hsa-miR-181a-3p |
| hsa-miR-151b_R+2 | hsa-miR-182-5p |
| hsa-miR-151a-5p | hsa-miR-183-5p |
| hsa-miR-151a-3p | hsa-miR-185-5p |
| hsa-miR-152-5p | hsa-miR-185-3p_R-1 |
| hsa-miR-152-3p | hsa-miR-186-5p_R+1 |
| hsa-miR-154-5p | hsa-miR-190b-5p |
| hsa-miR-154-3p | hsa-miR-191-5p |
| hsa-miR-155-5p_R-1 | hsa-miR-192-5p |
| hsa-miR-181a-5p | hsa-miR-193a-5p |
| hsa-miR-181a-2-3p | hsa-miR-193a-3p |
| hsa-miR-181b-5p_R+1 | hsa-miR-193b-5p |
| hsa-miR-181c-5p_R+2 | hsa-miR-193b-3p |
| hsa-miR-181c-3p_L-1R+1 | hsa-miR-194-5p |
| hsa-miR-181d-5p_R+1 | hsa-miR-194-5p_R+1 |
| hsa-miR-181a-3p | hsa-miR-195-5p_R+1 |
| hsa-miR-182-5p | hsa-miR-196b-5p |
| hsa-miR-183-5p | hsa-miR-196a-5p |
| hsa-miR-185-5p | hsa-miR-196a-3p_L+1R-1_1ss18CT |
| hsa-miR-185-3p_R-1 | hsa-miR-197-3p |
| hsa-miR-186-5p_R+1 | hsa-miR-199a-5p |
| hsa-miR-190a-5p_R+1 | hsa-miR-199b-3p_R-1 |
| hsa-miR-190b-5p | hsa-miR-199b-5p |
| hsa-miR-191-5p | hsa-miR-200c-3p |
| hsa-miR-192-5p | hsa-miR-200b-5p |
| hsa-miR-193a-5p | hsa-miR-200b-3p_R+1 |
| hsa-miR-193a-3p | hsa-miR-200a-5p |
| hsa-miR-193b-5p | hsa-miR-200a-3p_R+1 |
| hsa-miR-193b-3p | hsa-miR-203a-3p |
| hsa-miR-194-5p | hsa-miR-205-5p |
| hsa-miR-194-5p_R+1 | hsa-miR-205-3p_L-1R+1 |
| hsa-miR-195-5p_R+1 | hsa-miR-210-3p |
| hsa-miR-196b-5p | hsa-miR-212-3p_R+1 |
| hsa-miR-196a-5p | hsa-miR-215-5p_R+1 |
| hsa-miR-196a-3p_L+1R-1_1ss18CT | hsa-miR-218-5p_R+1 |
| hsa-miR-197-3p | hsa-miR-219a-5p_R+2 |
| hsa-miR-199a-5p | hsa-miR-221-5p_R+2 |
| hsa-miR-199b-3p_R-1 | hsa-miR-221-3p |
| hsa-miR-199b-5p | hsa-miR-222-3p_R+2 |
| hsa-miR-200c-3p | hsa-miR-224-5p_L-1R-2 |
| hsa-miR-200b-5p | hsa-miR-301b-3p |
| hsa-miR-200b-3p_R+1 | hsa-miR-301a-5p |
| hsa-miR-200a-5p | hsa-miR-301a-3p |
| hsa-miR-200a-3p_R+1 | hsa-miR-320d_R-1 |
| hsa-miR-203b-3p_R-1 | hsa-miR-320b_R-2 |
| hsa-miR-203a-3p | hsa-miR-320c_R-1 |
| hsa-miR-205-5p | hsa-miR-320a-3p |
| hsa-miR-205-3p_L-1R+1 | hsa-miR-323a-3p |
| hsa-miR-210-3p | hsa-miR-324-5p_R+1 |
| hsa-miR-212-5p | hsa-miR-324-3p_L-3R+1 |
| hsa-miR-212-3p_R+1 | hsa-miR-328-3p |
| hsa-miR-215-5p_R+1 | hsa-miR-330-5p_R-1 |
| hsa-miR-218-5p_R+1 | hsa-miR-330-3p |
| hsa-miR-219a-5p_R+2 | hsa-miR-331-5p_R-1 |
| hsa-miR-219b-5p | hsa-miR-331-3p |
| hsa-miR-221-5p_R+2 | hsa-miR-335-5p |
| hsa-miR-221-3p | hsa-miR-335-3p |
| hsa-mir-222-p5 | hsa-miR-339-5p_R-3 |
| hsa-miR-222-3p_R+2 | hsa-miR-339-3p |
| hsa-miR-224-5p_L-1R-2 | hsa-miR-340-5p |
| hsa-miR-299-3p | hsa-miR-342-3p_R+1 |
| hsa-miR-301b-3p | hsa-miR-345-5p |
| hsa-miR-301a-5p | hsa-miR-361-5p |
| hsa-miR-301a-3p | hsa-miR-361-3p |
| hsa-miR-320d_R-1 | hsa-miR-362-5p |
| hsa-miR-320b_R-2 | hsa-miR-365a-3p |
| hsa-miR-320c_R-1 | hsa-miR-370-3p |
| hsa-miR-320a-3p | hsa-miR-374a-5p |
| hsa-miR-323a-3p | hsa-miR-374a-3p |
| hsa-miR-324-5p_R+1 | hsa-miR-374b-5p |
| hsa-miR-324-3p_L-3R+1 | hsa-miR-374b-3p |
| hsa-miR-328-3p | hsa-miR-375-3p |
| hsa-miR-330-5p_R-1 | hsa-miR-376c-3p |
| hsa-miR-330-3p | hsa-miR-376a-3p |
| hsa-miR-331-5p_R-1 | hsa-miR-378a-5p |
| hsa-miR-331-3p | hsa-miR-378a-3p |
| hsa-miR-335-5p | hsa-miR-378d_1ss20AG |
| hsa-miR-335-3p | hsa-miR-378d_R-2 |
| hsa-miR-339-5p_R-3 | hsa-miR-378c_R-5 |
| hsa-miR-339-3p | hsa-miR-378i_R+1_1ss9AT |
| hsa-miR-340-5p | hsa-miR-379-5p |
| hsa-miR-342-5p_R+2 | hsa-miR-381-3p |
| hsa-miR-342-3p_R+1 | hsa-miR-382-5p |
| hsa-miR-345-5p | hsa-miR-382-3p_R+1 |
| hsa-miR-361-5p | hsa-miR-409-5p |
| hsa-miR-361-3p | hsa-miR-409-3p |
| hsa-miR-362-5p | hsa-miR-421 |
| hsa-miR-365a-3p | hsa-miR-423-5p |
| hsa-miR-365a-5p_R-1 | hsa-miR-423-3p |
| hsa-miR-369-3p | hsa-miR-424-5p_R-1 |
| hsa-miR-370-3p | hsa-miR-424-3p |
| hsa-miR-374a-5p | hsa-miR-425-5p |
| hsa-miR-374a-3p | hsa-miR-425-3p_L+1R-1 |
| hsa-miR-374b-5p | hsa-miR-429 |
| hsa-miR-374b-3p | hsa-miR-450a-5p |
| hsa-miR-375-3p | hsa-miR-450b-5p_R-1 |
| hsa-miR-376c-3p | hsa-miR-452-5p_R+1 |
| hsa-miR-376a-3p | mmu-miR-452-3p_1ss20GA |
| hsa-miR-378a-5p | hsa-miR-454-5p |
| hsa-miR-378a-3p | hsa-miR-454-3p_R+1 |
| hsa-miR-378d_1ss20AG | hsa-miR-455-5p |
| hsa-miR-378d_R-2 | hsa-miR-455-3p |
| hsa-miR-378c_R-5 | hsa-miR-484 |
| hsa-miR-378i_R+1_1ss9AT | hsa-miR-486-5p |
| hsa-miR-379-5p | hsa-miR-486-3p |
| hsa-miR-379-3p_R-1 | hsa-miR-487b-3p |
| hsa-miR-381-3p | hsa-miR-487a-3p |
| hsa-miR-382-5p | hsa-miR-493-5p |
| hsa-miR-382-3p_R+1 | hsa-miR-494-3p_R+1 |
| hsa-miR-409-5p | hsa-miR-495-3p |
| hsa-miR-409-3p | hsa-miR-497-5p_R+1 |
| hsa-miR-410-3p | hsa-miR-500a-5p_R+2 |
| hsa-miR-421 | hsa-miR-500a-3p_R+1 |
| hsa-miR-423-5p | hsa-miR-501-3p |
| hsa-miR-423-3p | hsa-miR-502-3p |
| hsa-miR-424-5p_R-1 | hsa-miR-503-5p_R-3 |
| hsa-miR-424-3p | hsa-miR-505-3p |
| hsa-miR-425-5p | hsa-miR-516a-5p_R-1 |
| hsa-miR-425-3p_L+1R-1 | hsa-miR-532-5p |
| hsa-miR-429 | hsa-miR-542-3p |
| hsa-miR-432-5p | hsa-miR-548k |
| hsa-miR-433-3p | hsa-miR-548o-3p |
| hsa-miR-450a-5p | hsa-miR-548bc_R+1 |
| hsa-miR-450a-1-3p | hsa-miR-548e-3p |
| hsa-miR-450b-5p_R-1 | hsa-miR-550a-3p |
| hsa-miR-450a-2-3p | hsa-miR-561-5p |
| hsa-miR-452-5p_R+1 | hsa-miR-574-5p |
| mmu-miR-452-3p_1ss20GA | hsa-miR-574-3p |
| hsa-miR-454-5p | hsa-miR-576-5p |
| hsa-miR-454-3p_R+1 | hsa-miR-576-3p |
| hsa-miR-455-5p | hsa-miR-582-5p |
| hsa-miR-455-3p | hsa-miR-582-3p |
| hsa-miR-484 | hsa-miR-584-5p_R-1 |
| hsa-miR-486-5p | hsa-miR-589-5p_R-1 |
| hsa-miR-486-3p | hsa-miR-590-5p |
| hsa-miR-487b-3p | hsa-miR-590-3p |
| hsa-miR-487a-3p | hsa-miR-597-5p |
| hsa-miR-491-5p | hsa-miR-615-3p_R-1 |
| hsa-miR-493-5p | hsa-miR-625-3p |
| hsa-miR-493-3p | hsa-miR-629-5p_R+1 |
| hsa-miR-494-3p_R+1 | hsa-miR-651-5p |
| hsa-miR-495-3p | hsa-miR-652-3p_R+1 |
| hsa-miR-497-5p_R+1 | hsa-miR-654-3p_R-2 |
| hsa-miR-499a-5p_R+1 | hsa-miR-660-5p_R+1 |
| hsa-miR-500a-5p_R+2 | hsa-mir-663a-p5 |
| hsa-miR-500a-3p_R+1 | hsa-mir-663a-p3 |
| hsa-miR-501-5p_R+2 | hsa-mir-663b-p3 |
| hsa-miR-501-3p | hsa-miR-664a-3p |
| hsa-miR-502-3p | hsa-miR-671-5p |
| hsa-miR-503-5p_R-3 | hsa-miR-708-5p |
| hsa-miR-505-3p | hsa-miR-708-3p_L-1R+1 |
| hsa-miR-516a-5p_R-1 | hsa-miR-744-5p |
| hsa-miR-532-5p | hsa-miR-744-3p_R-1 |
| hsa-miR-539-3p | hsa-miR-758-3p_R-1 |
| hsa-miR-542-3p | hsa-miR-769-5p |
| hsa-miR-548ad-5p_R+1 | hsa-miR-877-5p_R+3 |
| hsa-miR-548k | hsa-miR-889-3p |
| hsa-miR-548o-3p | hsa-miR-934 |
| hsa-miR-548bc_R+1 | hsa-miR-935_L-1 |
| hsa-miR-548e-3p | hsa-miR-940_R+1 |
| hsa-miR-550a-3p | hsa-miR-941 |
| hsa-miR-550a-5p_R-2 | hsa-miR-942-5p_L-2R+1 |
| hsa-mir-558-p5 | hsa-mir-944-p5 |
| hsa-miR-561-5p | hsa-miR-944 |
| hsa-miR-569_L-3_1ss20GC | hsa-miR-1180-3p |
| hsa-miR-574-5p | hsa-miR-1197 |
| hsa-miR-574-3p | hsa-miR-1246_R+1 |
| hsa-miR-576-5p | hsa-miR-1249-3p |
| hsa-miR-576-3p | hsa-miR-1260b_1ss9AG |
| hsa-miR-582-5p | hsa-mir-1268a-p3_1ss6GA |
| hsa-miR-582-3p | hsa-miR-1271-5p |
| hsa-miR-584-5p_R-1 | hsa-mir-1285-1-p5_1ss12AG |
| hsa-miR-589-5p_R-1 | hsa-miR-1287-5p |
| hsa-miR-589-3p_R-1 | hsa-miR-1290_1ss13TG |
| hsa-miR-590-5p | hsa-miR-1291_R-2 |
| hsa-miR-590-3p | hsa-miR-1296-5p_R-2 |
| hsa-miR-598-3p | hsa-miR-1301-3p_R-1 |
| hsa-miR-615-3p_R-1 | hsa-mir-1304-p5 |
| hsa-miR-619-5p_L-1R-2_1ss16AG | hsa-miR-1306-3p_R+4 |
| hsa-miR-625-5p_R-1 | hsa-miR-1307-5p |
| hsa-miR-625-3p | hsa-miR-1307-3p_R+1 |
| hsa-miR-629-5p_R+1 | rno-miR-1843b-5p_L+1R-2_1ss19AG |
| hsa-miR-651-5p | rno-miR-1843b-3p |
| hsa-miR-652-3p_R+1 | hsa-miR-1910-5p |
| hsa-miR-654-3p_R-2 | hsa-miR-2110_R-1 |
| hsa-miR-655-3p | hsa-miR-2355-5p_R+1 |
| hsa-miR-656-3p | hsa-miR-2355-3p_L-2R+2 |
| hsa-miR-660-5p_R+1 | hsa-miR-3065-5p |
| hsa-mir-663a-p5 | hsa-miR-3115 |
| hsa-mir-663a-p3 | hsa-miR-3177-3p |
| hsa-mir-663b-p3 | hsa-miR-3195_L+3R-1 |
| hsa-miR-664b-5p_R-1 | hsa-mir-3195-p3_1ss3CG |
| hsa-miR-664a-5p_R-2 | hsa-mir-3196-p5_1ss4GT |
| hsa-miR-664a-3p | hsa-miR-3615_R+1 |
| hsa-miR-671-5p | hsa-mir-3648-1-p5 |
| hsa-miR-671-3p_1ss21CT | hsa-mir-3648-1-p3 |
| hsa-miR-708-5p | hsa-mir-3665-p5_1ss17AG |
| hsa-miR-708-3p_L-1R+1 | hsa-mir-3929-p3_1ss16TG |
| hsa-miR-744-5p | hsa-miR-3934-5p |
| hsa-miR-744-3p_R-1 | hsa-mir-3960-p3_1ss16AT |
| hsa-miR-758-3p_R-1 | hsa-miR-4286_R+1 |
| hsa-miR-760_R+2 | hsa-miR-4326_R+4 |
| hsa-miR-766-3p | hsa-mir-4430-p3_1ss14AG |
| hsa-miR-769-5p | hsa-mir-4449-p3 |
| hsa-miR-769-3p_L-1 | hsa-mir-4454-p5 |
| hsa-miR-874-3p | hsa-miR-4485-3p_L+1R+1 |
| hsa-miR-877-5p_R+3 | hsa-miR-4488 |
| hsa-miR-877-3p_R+1 | hsa-miR-4492_L+1 |
| hsa-miR-889-3p | hsa-miR-4508_L+2 |
| hsa-miR-934 | hsa-miR-4516_L+1 |
| hsa-miR-935_L-1 | hsa-miR-4785 |
| hsa-miR-940_R+1 | hsa-mir-4787-p5_1ss6GC |
| hsa-miR-941 | hsa-mir-4791-p5_1ss3TC |
| hsa-miR-942-5p_L-2R+1 | hsa-miR-5010-3p_R+1 |
| hsa-mir-944-p5 | hsa-mir-6516-p5 |
| hsa-miR-944 | hsa-mir-7108-p3_1ss4GT |
| hsa-miR-1180-3p | hsa-mir-7110-p3_1ss18AC |
| hsa-miR-1185-1-3p | hsa-miR-7704_1ss19GC |
| hsa-miR-1197 | hsa-mir-9902-1-p3_1ss13GC |
| hsa-miR-1246_R+1 | hsa-miR-9985_R-1_1ss16CT |
| hsa-miR-1249-3p | hsa-mir-10396b-p5 |
| hsa-miR-1260b_1ss9AG | hsa-mir-10396b-p3 |
| hsa-mir-1268a-p3_1ss6GA | hsa-mir-10396a-p5 |
| hsa-miR-1271-5p | hsa-mir-10396a-p3 |
| hsa-mir-1273h-p5_1ss10GA | hsa-miR-10399-5p_R+1 |
| hsa-mir-1285-1-p5_1ss12AG | hsa-miR-10399-3p_L+1_1ss22CT |
| hsa-miR-1285-3p_R-2 | hsa-mir-10400-p5_2ss12GC19GA |
| hsa-miR-1287-5p | hsa-miR-10400-5p_R-3_1ss12GA |
| hsa-miR-1290_1ss13TG | hsa-miR-10400-5p_L-1R-2_1ss12GA |
| hsa-miR-1291_R-2 | hsa-miR-10400-5p_R-2_1ss12GA |
| hsa-mir-1291-p3 | hsa-mir-10401-p5 |
| hsa-miR-1296-5p_R-2 | hsa-mir-10401-p3 |
| hsa-miR-1301-3p_R-1 | hsa-miR-10527-5p_R+1 |
| hsa-mir-1303-p5_1ss18TA | hsa-miR-12136_R+8 |
| hsa-mir-1303-p3_1ss7GA | mmu-miR-326-3p |
| hsa-mir-1304-p5 | mml-mir-1235-p5_1ss7CT |
| hsa-miR-1304-3p_1ss13CA | cgr-mir-1285-p5_1ss6TC |
| hsa-miR-1307-5p | cgr-miR-1285_L-6 |
| hsa-miR-1307-3p_R+1 | ssc-mir-1285-p5 |
| rno-miR-1843b-5p_L+1R-2_1ss19AG | ssc-mir-1285-p3_1ss1GA |
| rno-miR-1843b-3p | mmr-miR-1839_L+1R-1 |
| hsa-miR-1910-5p | mmu-miR-1983 |
| hsa-miR-2110_R-1 | bta-miR-2424_L-2 |
| hsa-mir-2110-p3 | bta-mir-2904-1-p5 |
| hsa-miR-2355-5p_R+1 | bta-mir-2904-1-p3 |
| hsa-miR-2355-3p_L-2R+2 | mmu-miR-3535_R-3 |
| hsa-miR-3065-5p | cfa-miR-8903_R-2_1ss17CT |
| hsa-miR-3130-5p_R+1_1ss12CT | eca-mir-8986a-p5_1ss1GA |
| hsa-miR-3144-3p_R-1 | eca-mir-8986a-p3 |
| hsa-miR-3158-3p | efu-mir-9277-p3_1ss9CT |
| hsa-mir-3159-p3_1ss16TC | pal-miR-9298-5p_R+2 |
| hsa-miR-3177-3p | pal-mir-9298-p3 |
| hsa-miR-3187-3p | pal-mir-10001-p5_1ss19CT |
| hsa-miR-3195_L+3R-1 | mdo-miR-22-3p |
| hsa-mir-3195-p3_1ss3CG | mdo-miR-34b-5p |
| hsa-mir-3196-p5_1ss4GT | mmu-miR-92a-3p_R+1 |
| hsa-miR-3200-3p_R+1 | oga-miR-100_R+1 |
| hsa-miR-3615_R+1 | mmu-miR-146a-5p_R+1 |
| hsa-mir-3648-1-p5 | mdo-miR-181a-5p_R+3_2 |
| hsa-mir-3648-1-p3 | mdo-miR-200a-3p_R+2 |
| hsa-miR-3664-3p_R+1 | bta-miR-1246_L-1R+2 |
| hsa-mir-3665-p5_1ss17AG | mmu-mir-5126-p3_1ss14CT_2 |
| hsa-miR-3679-5p_R-4 | mmu-mir-5126-p3_1ss14CT_1 |
| hsa-miR-3909 | mmu-mir-6240-p5_1ss17GT |
| hsa-mir-3929-p5_1ss3AT | mmu-mir-6240-p5_1ss21TC |
| hsa-mir-3929-p3_1ss16TG | mmu-mir-6240-p5_1 |
| hsa-miR-3934-5p | eca-mir-8986b-p5_1ss1CG |
| hsa-mir-3960-p3_1ss16AT | efu-miR-9226_L-2R-2_1ss4AG |
| hsa-miR-4286_R+1 | bta-mir-12034-p3_1ss2TG_1 |
| hsa-miR-4326_R+4 | sha-mir-135a-p5_1ss6AG |
| hsa-mir-4430-p3_1ss14AG | mmu-miR-215-5p_R+1 |
| hsa-mir-4449-p3 | bta-miR-378_R+1 |
| hsa-mir-4454-p5 | bta-miR-1246_L-1R+3 |
| hsa-mir-4472-2-p3_1ss11AG | bta-miR-1246_R+2 |
| hsa-miR-4485-3p_L+1R+1 | bta-miR-1246_R+2_1ss18GN |
| hsa-miR-4488 | bta-miR-1246_L+1R-1 |
| hsa-miR-4492_L+1 | bta-miR-1246_L+1R+1_2 |
| hsa-miR-4508_L+2 | bta-miR-1246_R+3_1ss2AN |
| hsa-miR-4516_L+1 | bta-miR-1246_L+4R+1 |
| hsa-miR-4521_R+1 | bta-miR-1246_L+5R+2 |
| hsa-miR-4664-5p | bta-miR-1246_L+5R+1 |
| hsa-miR-4785 | bta-miR-1246_L+6 |
| hsa-mir-4787-p5_1ss6GC | bta-miR-1246_L+1R+3_1ss15AC |
| hsa-mir-4791-p5_1ss3TC | bta-miR-1246_L+1_1ss4TC |
| hsa-mir-5585-p3_1ss6AG | bta-miR-1246_R+2_1ss2AN |
| hsa-mir-5684-p3_1ss20CG | bta-miR-1246_R+2_1ss2AC |
| hsa-miR-6720-3p | bta-miR-1246_L+4R+2 |
| hsa-mir-7108-p3_1ss4GT | bta-mir-1246-p3_2ss5AC19AG |
| hsa-mir-7110-p3_1ss18AC | bta-miR-1246_L+4R+3 |
| hsa-miR-7704_1ss19GC | bta-miR-1246_L+1R+1_1 |
| hsa-miR-7706_R-1 | cgr-miR-1260 |
| hsa-miR-7851-3p | cja-mir-1302-p5_1ss7GC |
| hsa-miR-7976_R+3 | eca-mir-1543-p5 |
| hsa-mir-8086-p3_1ss13AG | eca-mir-1543-p3 |
| hsa-mir-9901-p5 | mmu-miR-2137_L-2_1ss16AG |
| hsa-mir-9902-1-p5_1ss4GA | mmu-miR-2137_L-2R-1_1ss16AG |
| hsa-mir-9902-1-p3_1ss13GC | mmu-miR-2137_L-3_1ss16AG |
| hsa-miR-9903 | bta-mir-2887-2-p5_1ss22AT |
| hsa-miR-9985_R-1_1ss16CT | bta-mir-2887-2-p3_1ss11AT_1 |
| hsa-miR-10395-3p_L+1 | bta-mir-2887-2-p3_1ss11AT_2 |
| hsa-mir-10396b-p5 | bta-mir-2887-2-p5_1ss11AT |
| hsa-mir-10396b-p3 | bta-mir-2887-2-p3_1ss10AT |
| hsa-mir-10396a-p5 | bta-mir-2887-2-p3_1ss5CA |
| hsa-mir-10396a-p3 | bta-mir-2887-2-p5_1ss2CT |
| hsa-miR-10399-5p_R+1 | bta-mir-2904-1-p5_1ss18TC |
| hsa-miR-10399-3p_L+1_1ss22CT | bta-mir-2887-2-p3_1ss2TC |
| hsa-mir-10400-p5_2ss12GC19GA | ssc-mir-4332-p3_1ss17CT |
| hsa-miR-10400-5p_R-3_1ss12GA | mmu-mir-5100-p3_1ss1AG |
| hsa-miR-10400-5p_L-1R-2_1ss12GA | mmu-mir-5106-p5_1ss15CT |
| hsa-miR-10400-5p_R-2_1ss12GA | mmu-miR-5126_R-2_1ss18CT |
| hsa-mir-10401-p3 | ssc-miR-4332_L-1R-1_1ss2AC |
| cja-miR-151_R+1 | mmu-mir-5126-p5_1ss15CT_1 |
| mmu-miR-326-3p | mmu-mir-5126-p5_1ss13CT_2 |
| mml-mir-1235-p5_1ss7CT | mmu-mir-5126-p5_1ss13CT_1 |
| cgr-mir-1285-p5_1ss6TC | mmu-miR-5126_L-1R-3_1ss18CT |
| cgr-miR-1285_L-6 | mmu-miR-5126_L-1R-2_1ss18CT |
| ssc-mir-1285-p5 | mmu-mir-5126-p5_1ss15CT_2 |
| ssc-mir-1285-p3_1ss1GA | mmu-mir-6236-p5_2 |
| mmr-miR-1839_L+1R-1 | mmu-mir-6236-p3_1ss23GC_2 |
| mmu-miR-1983 | mmu-mir-6236-p5_1 |
| bta-miR-2424_L-2 | mmu-mir-6236-p3_1ss23GC_1 |
| bta-mir-2904-1-p5 | mmu-mir-6236-p5_3 |
| bta-mir-2904-1-p3 | mmu-mir-6236-p3 |
| cja-mir-3135-p5_1ss12GT | mmu-mir-6236-p3_1ss16GC |
| cja-mir-3135-p3_1ss14CA | mmu-mir-6236-p3_1ss21GA |
| mmu-miR-3535_R-3 | mmu-miR-6239_R-2_1ss6TG |
| mmu-mir-3535-p3 | mmu-mir-6240-p5_1ss14TG |
| mmu-miR-7689-3p | mmu-mir-6240-p3_1ss11TG |
| eca-mir-8986a-p5_1ss1GA | mmu-mir-6240-p5_3 |
| eca-mir-8986a-p3 | mmu-mir-6240-p5_1ss15GT |
| efu-mir-9277-p5 | mmu-mir-6240-p5_1ss20TC |
| efu-mir-9277-p3_1ss9CT | mmu-mir-6240-p5_2 |
| pal-miR-9298-5p_R+2 | mmu-mir-6345-p5_1ss14AG |
| pal-mir-9298-p3 | mmu-mir-8112-p5_1ss13CT |
| pal-mir-10001-p5_1ss19CT | mmu-mir-8119-p3_1ss4GT |
| mdo-miR-22-3p | cja-mir-9976-2-p5_1ss12TG |
| mdo-miR-34b-5p | bta-miR-11980_R-2_1ss4CG |
| mmu-miR-92a-3p_R+1 | bta-miR-11987_L-2_1ss8TA |
| oga-miR-100_R+1 | bta-miR-11987_L-2R-1_1ss8TA |
| mmu-miR-146a-5p_R+1 | bta-miR-11987_L-2_1ss8TC |
| cja-miR-151_R+2 | bta-mir-11987-p3_1ss6TA |
| mdo-miR-181a-5p_R+3_2 | bta-miR-11987_L-1R+1_1ss8TA |
| dno-miR-196c-5p | bta-miR-11987_L-1_1ss8TA |
| mdo-miR-200a-3p_R+2 | bta-miR-11987_L-2R-1_1ss8TC |
| ssc-miR-339_R+1 | bta-miR-12034_L+1R-2 |
| nle-miR-378d_R+2 | bta-mir-12034-p3_1ss2TG_2 |
| mmu-miR-455-5p_L-2R+3 | mmu-miR-92a-3p_R+2 |
| bta-miR-1246_L-1R+2 | oga-miR-100_R+2 |
| mmu-mir-6240-p5_1ss17GT | mdo-miR-181a-5p_R+3_1 |
| mmu-mir-6240-p5_1ss21TC | bta-miR-378_R+2 |
| mmu-mir-6240-p5_1 | bta-miR-1246_L+1R-2 |
| eca-mir-8986b-p5_1ss1CG | mmu-miR-5106_L-5_1ss20CT |
| efu-miR-9226_L-2R-2_1ss4AG | mmu-miR-5126_L-4_1ss18CT |
| ssc-mir-9786-1-p3_1ss2TA | efu-miR-9341_L-3R+1 |
| bta-mir-12034-p3_1ss2TG_1 | cja-miR-9984_1ss4CG |
| mmu-let-7j_R-2 | bta-miR-11975_L-2_1ss14CG |
| tch-let-7a-5p | mmu-let-7j_1ss8TG |
| bta-miR-339b_R+3 |  |
| mmu-miR-378d_R-1 |  |
| bta-miR-378_R+1 |  |
| bta-miR-378c |  |
| bta-miR-1246_L-1R+3 |  |
| bta-miR-1246_R+3_1ss4GT |  |
| bta-miR-1246_R+2 |  |
| bta-miR-1246_R+2_1ss18GN | |
| bta-miR-1246_L+1R-1 |  |
| bta-miR-1246_L+1R+1_2 |  |
| bta-miR-1246_R+3_1ss2AN |  |
| bta-miR-1246_L+4R+1 |  |
| bta-miR-1246_L+5R+2 |  |
| bta-miR-1246_L+5R+1 |  |
| bta-miR-1246_L+1R+3_1ss15AC | |
| bta-miR-1246_L+1_1ss4TC |  |
| bta-miR-1246_R+2_1ss2AN |  |
| bta-miR-1246_R+2_1ss2AC |  |
| bta-miR-1246_L+4R+2 |  |
| bta-mir-1246-p3_2ss5AC19AG | |
| bta-miR-1246_L+4R+3 |  |
| bta-miR-1246_L+1R+1_1 |  |
| cgr-miR-1260 |  |
| cja-mir-1302-p5_1ss7GC |  |
| eca-mir-1543-p5 |  |
| eca-mir-1543-p3 |  |
| mmu-miR-2137_L-2_1ss16AG | |
| mmu-miR-2137_L-2R-1_1ss16AG | |
| mmu-miR-2137_L-3_1ss16AG | |
| bta-mir-2887-2-p5_1ss22AT |  |
| bta-mir-2887-2-p3_1ss11AT_1 | |
| bta-mir-2887-2-p3_1ss11AT_2 | |
| bta-mir-2887-2-p5_1ss11AT |  |
| bta-mir-2887-2-p3_1ss10AT |  |
| bta-mir-2887-2-p3_1ss5CA |  |
| bta-mir-2887-2-p5_1ss2CT |  |
| bta-mir-2904-1-p5_1ss18TC |  |
| mmu-mir-3969-p3_1ss13TC |  |
| mmu-miR-3969_L-2_1ss15TC | |
| ssc-mir-4332-p3_1ss17CT |  |
| mmu-mir-5100-p3_1ss1AG |  |
| mmu-mir-5106-p5_1ss15CT |  |
| mmu-miR-5126_R-2_1ss18CT | |
| ssc-miR-4332_L-1R-1_1ss2AC | |
| mmu-mir-5126-p5_1ss15CT_1 | |
| mmu-mir-5126-p5_1ss13CT_2 | |
| mmu-mir-5126-p5_1ss13CT_1 | |
| mmu-miR-5126_L-1R-3_1ss18CT | |
| mmu-miR-5126_L-1R-2_1ss18CT | |
| mmu-mir-5126-p5_1ss15CT_2 | |
| mmu-mir-6236-p5_2 |  |
| mmu-mir-6236-p3_1ss23GC_2 | |
| mmu-mir-6236-p5_1 |  |
| mmu-mir-6236-p3_1ss23GC_1 | |
| mmu-mir-6236-p5_3 |  |
| mmu-mir-6236-p3 |  |
| mmu-mir-6236-p3_1ss16GC |  |
| mmu-mir-6236-p3_1ss21GA |  |
| mmu-miR-6239_R-2_1ss6TG | |
| mmu-mir-6240-p5_1ss14TG |  |
| mmu-mir-6240-p3_1ss11TG |  |
| mmu-mir-6240-p5_3 |  |
| mmu-mir-6240-p5_1ss15GT |  |
| mmu-mir-6240-p5_1ss20TC |  |
| mmu-mir-6240-p5_2 |  |
| mmu-mir-6345-p5_1ss14AG |  |
| mml-mir-7182-p3_1ss10CG_2 | |
| mml-mir-7182-p3_1ss10CG_1 | |
| mmu-mir-8112-p5_1ss13CT |  |
| cja-miR-9933_L-4_1ss12AT |  |
| cja-mir-9976-2-p5_1ss12TG |  |
| cja-mir-9984-2-p3_1ss10CT |  |
| cja-mir-9984-2-p5_1ss10CT |  |
| cja-mir-9984-2-p3_1ss7CT |  |
| bta-miR-11980_R-2_1ss4CG |  |
| bta-miR-11987_L-2_1ss8TA |  |
| bta-miR-11987_L-2R-1_1ss8TA | |
| bta-miR-11987_L-2_1ss8TC |  |
| bta-mir-11987-p3_1ss6TA |  |
| bta-miR-11987_L-1R+1_1ss8TA | |
| bta-miR-11987_L-1_1ss8TA |  |
| bta-miR-11987_L-2R-1_1ss8TC | |
| bta-miR-12034_L+1R-2 |  |
| bta-mir-12034-p3_1ss2TG_2 |  |
| mmu-miR-92a-3p_R+2 |  |
| oga-miR-100_R+2 |  |
| sha-miR-125a_R+2 |  |
| mdo-miR-181a-5p_R+3_1 |  |
| mdo-miR-181b-5p_R+3 |  |
| mdo-miR-200a-3p_R+3 |  |
| bta-miR-378_R+2 |  |
| mmu-miR-1195_L-1R-4_1ss11GA | |
| bta-miR-1246_L+1R-2 |  |
| mmu-miR-5106_L-5_1ss20CT | |
| mmu-miR-5126_L-4_1ss18CT | |
| efu-miR-9341_L-3R+1 |  |
| cja-miR-9984_1ss4CG |  |
| bta-miR-11975_L-2_1ss14CG | |
| mmu-let-7j_1ss8TG |  |
