# Supplementary material for: Association of Porphyromonas gingivalis-infected oral squamous cell carcinoma cell-secreted exosomal miR-3648-1-p5 with tumor progression
Source: Cancer Cell Int. 2026 Mar 4;26:163. doi: 10.1186/s12935-026-04230-5 (PMC13069696; doi:10.1186/s12935-026-04230-5)
Supplement: Supplementary file 5 — Supplementary Material 5 [file 12935_2026_4230_MOESM5_ESM.docx]

**Additional File 5. miRNAs with significantly different expression levels between Ex and *Pg*.Ex**

| miR_name | log2(FC) | pvalue(t_test) |
| --- | --- | --- |
| hsa-mir-3648-1-p3 | -1.79 | 0.0000047270 |
| mmu-mir-6240-p5_1ss17GT | 1.19 | 0.0000075146 |
| bta-mir-2904-1-p5 | 1.33 | 0.0000175187 |
| mmu-miR-5126_R-2_1ss18CT | 0.76 | 0.0000213111 |
| mmu-mir-6240-p5_1ss14TG | 1.36 | 0.000112268 |
| hsa-miR-7704_1ss19GC | 1.99 | 0.000117189 |
| mmu-mir-6236-p3_1ss23GC_2 | 0.93 | 0.000145885 |
| mmu-mir-6236-p3_1ss23GC_1 | 0.93 | 0.000145885 |
| bta-mir-2887-2-p3_1ss11AT_1 | 1.79 | 0.00014759 |
| bta-mir-2887-2-p3_1ss11AT_2 | 1.79 | 0.00014759 |
| bta-mir-2887-2-p5_1ss11AT | 1.79 | 0.00014759 |
| hsa-mir-3648-1-p5 | -1.43 | 0.00029967 |
| hsa-miR-3195_L+3R-1 | 2.24 | 0.000409063 |
| hsa-miR-183-5p | -0.23 | 0.000516125 |
| mmu-miR-2137_L-2_1ss16AG | 1.03 | 0.000593918 |
| bta-mir-12034-p3_1ss2TG_1 | 1.25 | 0.000650286 |
| bta-mir-12034-p3_1ss2TG_2 | 1.25 | 0.000650286 |
| bta-mir-2887-2-p5_1ss22AT | 1.69 | 0.000844429 |
| hsa-miR-200a-3p_R+1 | -0.23 | 0.00105859 |
| mmu-mir-6240-p5_1 | 1.16 | 0.00119483 |
| mmu-mir-6240-p5_3 | 1.16 | 0.00119483 |
| mmu-mir-6240-p5_2 | 1.16 | 0.00119483 |
| hsa-miR-935_L-1 | -0.60 | 0.001373979 |
| hsa-miR-301a-5p | -3.13 | 0.001546429 |
| bta-miR-11980_R-2_1ss4CG | 0.82 | 0.001551626 |
| mmu-mir-6240-p3_1ss11TG | 1.22 | 0.001685691 |
| hsa-miR-7-5p | 0.51 | 0.002022687 |
| mmu-miR-2137_L-3_1ss16AG | 0.73 | 0.002174604 |
| hsa-miR-140-3p_L-1R+2 | -0.47 | 0.002182618 |
| mmu-miR-2137_L-2R-1_1ss16AG | 0.82 | 0.002545835 |
| mmu-miR-5126_L-1R-3_1ss18CT | 0.71 | 0.002771493 |
| hsa-miR-139-5p | -0.86 | 0.002922871 |
| bta-mir-2904-1-p3 | 1.01 | 0.003104383 |
| mmu-mir-6236-p5_2 | 0.39 | 0.00346242 |
| mmu-mir-6236-p5_1 | 0.39 | 0.00346242 |
| mmu-mir-6236-p5_3 | 0.39 | 0.00346242 |
| mmu-miR-5126_L-1R-2_1ss18CT | 0.74 | 0.003635691 |
| hsa-miR-33a-3p_R-1 | -2.79 | 0.004160377 |
| hsa-miR-148b-3p | -0.56 | 0.004196614 |
| hsa-miR-30d-3p_L-1 | -2.75 | 0.004463203 |
| hsa-mir-10396b-p3 | -1.98 | 0.004489416 |
| hsa-mir-10396a-p3 | -1.98 | 0.004489416 |
| hsa-miR-31-5p_R+1 | -0.28 | 0.005179565 |
| hsa-miR-185-3p_R-1 | -1.56 | 0.005453072 |
| mmu-miR-5106_L-5_1ss20CT | -0.94 | 0.005508972 |
| hsa-miR-221-5p_R+2 | -0.45 | 0.005585571 |
| hsa-miR-708-5p | -0.60 | 0.005843309 |
| bta-mir-2887-2-p3_1ss5CA | -0.75 | 0.005911161 |
| mmu-mir-6240-p5_1ss15GT | 1.36 | 0.005937339 |
| mmu-miR-6239_R-2_1ss6TG | 2.07 | 0.006055787 |
| hsa-mir-1285-1-p5_1ss12AG | -4.83 | 0.006096026 |
| bta-miR-11987_L-2R-1_1ss8TA | 0.69 | 0.00661799 |
| mmu-mir-6236-p3_1ss16GC | 1.16 | 0.006689696 |
| hsa-miR-151a-3p | 0.17 | 0.006713176 |
| bta-miR-1246_L+1R-2 | 1.99 | 0.008189976 |
| hsa-mir-10396b-p5 | -4.69 | 0.00832856 |
| hsa-mir-10396a-p5 | -4.69 | 0.00832856 |
| hsa-mir-4449-p3 | -3.45 | 0.008363667 |
| pal-mir-10001-p5_1ss19CT | -0.85 | 0.009018505 |
| hsa-miR-494-3p_R+1 | 0.97 | 0.00910352 |
| hsa-miR-34b-3p_L-1R+1 | -2.60 | 0.009254868 |
| hsa-miR-561-5p | -0.72 | 0.009409826 |
| ssc-mir-1285-p5 | 1.60 | 0.010613558 |
| hsa-miR-1246_R+1 | 0.82 | 0.011339359 |
| hsa-miR-182-5p | 0.14 | 0.013635183 |
| hsa-mir-3929-p3_1ss16TG | -1.04 | 0.014037178 |
| hsa-miR-497-5p_R+1 | -2.72 | 0.014178236 |
| hsa-miR-17-3p | 0.64 | 0.015023774 |
| efu-miR-9226_L-2R-2_1ss4AG | 0.95 | 0.016016378 |
| hsa-miR-4508_L+2 | 0.59 | 0.016297336 |
| bta-miR-1246_L+1R-1 | 2.34 | 0.016495968 |
| eca-mir-8986a-p5_1ss1GA | 0.90 | 0.017223019 |
| hsa-miR-126-3p_R-1 | -0.81 | 0.017511945 |
| hsa-miR-4492_L+1 | 0.45 | 0.017986974 |
| hsa-miR-30e-5p_R+2 | -0.67 | 0.01830061 |
| hsa-miR-425-3p_L+1R-1 | -1.04 | 0.018348735 |
| hsa-miR-30d-5p_R+2 | -0.48 | 0.018545851 |
| cgr-miR-1260 | -3.61 | 0.019120685 |
| hsa-miR-149-5p_R-3 | -0.29 | 0.019175225 |
| hsa-miR-132-5p | -2.29 | 0.021766767 |
| hsa-miR-362-5p | -1.21 | 0.022442507 |
| hsa-let-7f-1-3p_1ss22CT | -2.60 | 0.022615598 |
| hsa-miR-127-3p | -0.90 | 0.022718548 |
| hsa-let-7b-5p | -0.47 | 0.02294821 |
| hsa-miR-30a-5p_R+2 | -0.31 | 0.023095785 |
| bta-miR-1246_R+2 | 0.65 | 0.023457448 |
| hsa-miR-27b-5p | 0.81 | 0.023704376 |
| hsa-miR-4516_L+1 | -0.47 | 0.024072654 |
| bta-miR-378_R+1 | 0.22 | 0.024979831 |
| cja-miR-9984_1ss4CG | -0.40 | 0.025186292 |
| hsa-miR-193a-5p | -0.83 | 0.025212583 |
| hsa-miR-30b-5p | 0.28 | 0.026162276 |
| hsa-mir-4791-p5_1ss3TC | -0.64 | 0.026921692 |
| hsa-miR-15b-3p_R-1 | 0.25 | 0.027295941 |
| mmu-mir-6236-p3_1ss21GA | -2.36 | 0.027482545 |
| hsa-miR-195-5p_R+1 | -2.27 | 0.027629056 |
| bta-mir-2904-1-p5_1ss18TC | 1.87 | 0.028126721 |
| mmu-mir-5126-p5_1ss13CT_2 | 1.37 | 0.028766632 |
| mmu-mir-5126-p5_1ss13CT_1 | 1.37 | 0.028766632 |
| hsa-miR-34c-3p | -2.20 | 0.028891174 |
| hsa-miR-934 | -1.26 | 0.029357921 |
| hsa-miR-708-3p_L-1R+1 | -0.77 | 0.030315392 |
| hsa-miR-4286_R+1 | 0.91 | 0.032170314 |
| hsa-miR-210-3p | -0.48 | 0.033132875 |
| hsa-let-7i-5p | -0.19 | 0.034257734 |
| hsa-miR-95-3p_R-1 | -0.71 | 0.036328659 |
| hsa-miR-374a-5p | -0.45 | 0.036801266 |
| hsa-miR-671-5p | -1.40 | 0.037774232 |
| hsa-miR-26b-5p_R+1 | -0.10 | 0.038442457 |
| hsa-miR-193a-3p | -0.93 | 0.039351828 |
| hsa-miR-590-3p | -0.77 | 0.039521338 |
| hsa-miR-151b_R+2 | -0.16 | 0.039624955 |
| hsa-mir-3195-p3_1ss3CG | 1.08 | 0.040248374 |
| hsa-miR-331-3p | -0.58 | 0.041090035 |
| hsa-miR-629-5p_R+1 | -0.34 | 0.043610931 |
| hsa-mir-7108-p3_1ss4GT | 0.95 | 0.043993781 |
| hsa-miR-941 | -0.35 | 0.044747196 |
| hsa-miR-106b-3p | 0.28 | 0.044809035 |
| hsa-miR-200b-5p | -0.51 | 0.045462853 |
| hsa-mir-1304-p5 | -2.03 | 0.046059606 |
| hsa-miR-324-5p_R+1 | -0.36 | 0.046601087 |
| hsa-miR-181a-5p | -0.18 | 0.048755474 |
| hsa-miR-30c-5p_R+1 | 0.30 | 0.050048471 |
| mmu-miR-5126_L-4_1ss18CT | 0.92 | 0.050665912 |
| hsa-miR-194-5p_R+1 | -0.23 | 0.051629796 |
| hsa-miR-28-5p | -0.49 | 0.052969802 |
| bta-miR-1246_L-1R+3 | 0.48 | 0.053123679 |
| hsa-miR-27a-3p_R-1 | -0.25 | 0.053432501 |
| hsa-miR-186-5p_R+1 | 0.37 | 0.053481938 |
| hsa-miR-96-5p | -0.40 | 0.055676805 |
| eca-mir-1543-p3 | -1.98 | 0.056484533 |
| hsa-miR-1296-5p_R-2 | -0.49 | 0.05757679 |
| ssc-mir-1285-p3_1ss1GA | -0.51 | 0.060164901 |
| ssc-mir-4332-p3_1ss17CT | 1.04 | 0.062565136 |
| hsa-miR-98-5p | -0.27 | 0.062630848 |
| hsa-miR-34b-5p_L-1R+1 | -1.93 | 0.062690167 |
| hsa-miR-375-3p | -0.57 | 0.064500263 |
| hsa-miR-99b-5p | 0.22 | 0.066364686 |
| hsa-let-7a-5p | -0.37 | 0.067075957 |
| hsa-miR-99a-5p_R-1 | -1.36 | 0.068781009 |
| hsa-let-7f-2-3p_1ss22CT | -0.70 | 0.070810282 |
| hsa-miR-361-3p | -0.57 | 0.071882059 |
| hsa-miR-574-3p | 0.14 | 0.075274313 |
| hsa-mir-4787-p5_1ss6GC | -0.79 | 0.082294382 |
| hsa-miR-218-5p_R+1 | -2.47 | 0.086062974 |
| hsa-miR-29b-3p | -0.58 | 0.087557119 |
| hsa-miR-30a-3p | -0.14 | 0.088006215 |
| hsa-miR-500a-3p_R+1 | -0.89 | 0.09001981 |
| hsa-miR-370-3p | -1.61 | 0.090899991 |
| hsa-miR-9985_R-1_1ss16CT | -1.76 | 0.092447742 |
| mdo-miR-22-3p | -0.61 | 0.093687351 |
| hsa-miR-548e-3p | -1.75 | 0.095269472 |
| hsa-miR-3065-5p | -0.55 | 0.098603731 |
| hsa-miR-10400-5p_R-2_1ss12GA | -1.73 | 0.099751146 |
| hsa-miR-500a-5p_R+2 | -1.93 | 0.10525045 |
| hsa-miR-1307-3p_R+1 | -0.24 | 0.105661469 |
| hsa-miR-625-3p | -0.65 | 0.1084467 |
| hsa-miR-31-3p_R+1 | -0.24 | 0.108732128 |
| hsa-miR-30c-2-3p | -0.58 | 0.109230006 |
| hsa-miR-944 | 0.18 | 0.109322664 |
| hsa-miR-374b-5p | -0.46 | 0.110743432 |
| cja-mir-9976-2-p5_1ss12TG | -1.92 | 0.110872102 |
| bta-miR-1246_L+4R+1 | 1.39 | 0.112725392 |
| hsa-miR-1249-3p | -1.96 | 0.112945185 |
| hsa-miR-34a-5p | -0.65 | 0.113469948 |
| hsa-miR-136-3p | -1.66 | 0.117239309 |
| hsa-miR-199a-5p | -1.38 | 0.118458687 |
| hsa-miR-199b-3p_R-1 | -1.16 | 0.118846126 |
| hsa-miR-503-5p_R-3 | -0.12 | 0.118850495 |
| bta-miR-1246_L+4R+3 | 1.05 | 0.120177351 |
| hsa-miR-148a-3p | -0.10 | 0.121816199 |
| hsa-mir-944-p5 | -1.15 | 0.122373387 |
| hsa-miR-16-5p | -0.31 | 0.122911759 |
| hsa-miR-584-5p_R-1 | -0.27 | 0.123459982 |
| hsa-mir-663a-p5 | -1.98 | 0.125175712 |
| hsa-mir-10401-p3 | 1.15 | 0.125930805 |
| hsa-miR-484 | -0.23 | 0.127410787 |
| hsa-miR-205-3p_L-1R+1 | -1.63 | 0.127551342 |
| hsa-miR-4488 | -0.44 | 0.128914622 |
| eca-mir-1543-p5 | 0.44 | 0.129366812 |
| hsa-miR-2110_R-1 | -1.19 | 0.130882922 |
| hsa-miR-1307-5p | -0.17 | 0.132167068 |
| bta-miR-11987_L-2R-1_1ss8TC | -1.62 | 0.132826352 |
| hsa-miR-203a-3p | 0.24 | 0.133649247 |
| hsa-miR-744-3p_R-1 | -1.27 | 0.137344693 |
| hsa-miR-2355-5p_R+1 | 0.70 | 0.13750358 |
| hsa-miR-4785 | -1.63 | 0.138241153 |
| hsa-miR-15b-5p | -0.21 | 0.138305569 |
| hsa-miR-429 | -0.21 | 0.139729624 |
| hsa-miR-330-3p | -0.31 | 0.140635032 |
| hsa-miR-374a-3p | -0.31 | 0.1414435 |
| hsa-miR-542-3p | -0.38 | 0.142253748 |
| hsa-miR-34c-5p | -0.14 | 0.144821855 |
| hsa-miR-200a-5p | -0.47 | 0.14617689 |
| mmu-let-7j_1ss8TG | -0.74 | 0.146526129 |
| bta-miR-1246_L+5R+2 | 0.52 | 0.147377795 |
| hsa-miR-212-3p_R+1 | -2.24 | 0.153595281 |
| hsa-miR-24-3p_R-2 | -0.06 | 0.154654819 |
| hsa-miR-450b-5p_R-1 | 0.12 | 0.163642019 |
| hsa-miR-222-3p_R+2 | -0.25 | 0.163848959 |
| hsa-miR-130b-5p_R+1 | -1.09 | 0.168718124 |
| mml-mir-1235-p5_1ss7CT | -2.35 | 0.170622387 |
| mmu-miR-3535_R-3 | 1.14 | 0.175758066 |
| hsa-miR-3934-5p | 0.25 | 0.17821978 |
| hsa-miR-10400-5p_L-1R-2_1ss12GA | -1.94 | 0.183328687 |
| hsa-miR-197-3p | 0.16 | 0.183984889 |
| hsa-miR-125b-5p | -0.19 | 0.186390013 |
| hsa-miR-103a-3p | 0.15 | 0.187363515 |
| bta-miR-11975_L-2_1ss14CG | -2.34 | 0.187708469 |
| hsa-miR-221-3p | -0.23 | 0.187948942 |
| hsa-miR-129-1-3p | 0.26 | 0.19173054 |
| hsa-miR-17-5p | 0.16 | 0.191903868 |
| hsa-miR-106a-5p_1ss1AC | 0.16 | 0.191903868 |
| hsa-miR-505-3p | -0.86 | 0.193348451 |
| hsa-miR-19b-3p | 0.23 | 0.194132799 |
| bta-miR-1246_R+3_1ss2AN | 0.88 | 0.19627854 |
| hsa-miR-574-5p | -0.28 | 0.200831372 |
| hsa-miR-582-3p | -1.61 | 0.202714153 |
| hsa-miR-141-3p_R+1 | -0.28 | 0.203312949 |
| hsa-miR-330-5p_R-1 | 1.32 | 0.204057117 |
| hsa-miR-342-3p_R+1 | 0.17 | 0.204217393 |
| hsa-miR-129-5p | -0.28 | 0.206238711 |
| mmr-miR-1839_L+1R-1 | -0.16 | 0.2121596 |
| bta-miR-12034_L+1R-2 | -0.43 | 0.212534618 |
| hsa-mir-1268a-p3_1ss6GA | -0.80 | 0.213847721 |
| hsa-miR-27b-3p | -0.08 | 0.217112816 |
| hsa-miR-376a-3p | -1.37 | 0.217947614 |
| hsa-miR-324-3p_L-3R+1 | -0.48 | 0.218940618 |
| hsa-miR-10b-5p_R-1 | -0.19 | 0.221220963 |
| hsa-miR-181a-3p | 0.23 | 0.224295993 |
| hsa-miR-15a-5p_R-1 | -0.37 | 0.225081781 |
| hsa-miR-487a-3p | -1.54 | 0.225660913 |
| hsa-miR-155-5p_R-1 | 0.14 | 0.228935398 |
| hsa-miR-93-3p_R+1 | -0.90 | 0.229030061 |
| hsa-miR-502-3p | -0.74 | 0.229599404 |
| hsa-miR-181d-5p_R+1 | 0.17 | 0.230139792 |
| mdo-miR-181a-5p_R+3_2 | -0.43 | 0.230596599 |
| mdo-miR-181a-5p_R+3_1 | -0.43 | 0.230596599 |
| hsa-miR-382-5p | -1.02 | 0.231918509 |
| hsa-miR-101-3p_R+1 | -0.38 | 0.233493874 |
| hsa-miR-26a-5p | -0.15 | 0.234872918 |
| hsa-mir-7110-p3_1ss18AC | 0.30 | 0.235154806 |
| mmu-miR-452-3p_1ss20GA | -0.25 | 0.235870551 |
| hsa-miR-424-5p_R-1 | -0.19 | 0.238120636 |
| hsa-miR-455-3p | -0.12 | 0.239266132 |
| mmu-miR-326-3p | -0.97 | 0.240840552 |
| eca-mir-8986b-p5_1ss1CG | 0.40 | 0.246853876 |
| hsa-let-7c-5p | -0.38 | 0.248978868 |
| hsa-miR-425-5p | -0.28 | 0.251006703 |
| hsa-miR-423-5p | -0.11 | 0.253230666 |
| mmu-miR-1983 | -0.44 | 0.255262215 |
| hsa-miR-148b-5p_L+1 | -1.80 | 0.25917838 |
| hsa-miR-409-3p | -1.12 | 0.259273054 |
| bta-mir-2887-2-p3_1ss10AT | 0.60 | 0.260611753 |
| hsa-miR-1260b_1ss9AG | -0.78 | 0.260990519 |
| hsa-miR-424-3p | 0.23 | 0.261874231 |
| hsa-miR-493-5p | -0.47 | 0.266926125 |
| hsa-miR-589-5p_R-1 | 0.26 | 0.269252834 |
| hsa-miR-501-3p | -0.30 | 0.279579048 |
| hsa-mir-3665-p5_1ss17AG | 0.25 | 0.282084109 |
| hsa-miR-744-5p | 0.14 | 0.284864054 |
| bta-miR-1246_L+1R+3_1ss15AC | -2.09 | 0.287432894 |
| cgr-mir-1285-p5_1ss6TC | 0.34 | 0.289098817 |
| hsa-miR-7-1-3p | -1.34 | 0.290148527 |
| hsa-miR-20a-3p_L-1R+1 | -1.22 | 0.294948617 |
| hsa-miR-582-5p | -1.33 | 0.295547061 |
| hsa-miR-215-5p_R+1 | -1.35 | 0.295625725 |
| hsa-let-7d-5p | -0.16 | 0.305883278 |
| hsa-miR-192-5p | -0.08 | 0.311507415 |
| hsa-miR-125a-5p_R-1 | -0.17 | 0.31215091 |
| hsa-miR-199b-5p | -1.17 | 0.313543713 |
| mmu-mir-5106-p5_1ss15CT | -0.52 | 0.314734254 |
| hsa-miR-335-3p | -0.20 | 0.315307275 |
| hsa-miR-452-5p_R+1 | -0.16 | 0.318642988 |
| hsa-miR-100-5p | -0.12 | 0.319854905 |
| hsa-miR-548bc_R+1 | -1.30 | 0.320149409 |
| hsa-miR-140-5p | 0.21 | 0.32768752 |
| hsa-miR-889-3p | 0.41 | 0.328688348 |
| bta-miR-1246_L-1R+2 | 0.22 | 0.329316394 |
| hsa-let-7a-3p | -0.80 | 0.335290847 |
| hsa-miR-9-5p | -0.14 | 0.337136714 |
| efu-miR-9341_L-3R+1 | 0.45 | 0.338614616 |
| hsa-miR-10a-5p_R-1 | -0.07 | 0.340903228 |
| hsa-miR-135b-5p | -0.12 | 0.340985344 |
| hsa-miR-495-3p | 0.38 | 0.352345502 |
| hsa-miR-1287-5p | -0.35 | 0.355876518 |
| hsa-let-7f-5p | -0.17 | 0.356610209 |
| hsa-miR-301a-3p | -0.24 | 0.357963818 |
| hsa-miR-320d_R-1 | -0.67 | 0.358170436 |
| hsa-miR-345-5p | 0.26 | 0.358850825 |
| bta-miR-11987_L-1_1ss8TA | -0.14 | 0.362446662 |
| hsa-miR-92a-3p | 0.15 | 0.363254941 |
| hsa-miR-365a-3p | -0.13 | 0.363351778 |
| hsa-miR-22-3p | -0.19 | 0.365108676 |
| hsa-miR-10400-5p_R-3_1ss12GA | -0.14 | 0.368985465 |
| hsa-miR-194-5p | -0.14 | 0.369241647 |
| mmu-mir-6345-p5_1ss14AG | -0.28 | 0.370711601 |
| hsa-miR-1180-3p | 0.21 | 0.37285032 |
| bta-miR-1246_L+5R+1 | 0.45 | 0.379167147 |
| hsa-let-7d-3p | -0.17 | 0.379238183 |
| hsa-miR-376c-3p | -0.70 | 0.381166869 |
| hsa-miR-455-5p | 0.13 | 0.384217475 |
| bta-miR-1246_R+2_1ss2AN | -0.77 | 0.384639227 |
| hsa-miR-421 | 0.28 | 0.384681392 |
| hsa-miR-196a-3p_L+1R-1_1ss18CT | -0.57 | 0.385254333 |
| hsa-miR-143-3p_R+1 | -0.60 | 0.3928211 |
| hsa-miR-1197 | 1.41 | 0.393113987 |
| hsa-miR-128-3p | -0.13 | 0.394708902 |
| hsa-let-7a-3p_R+1 | -0.26 | 0.401094431 |
| efu-mir-9277-p3_1ss9CT | -0.45 | 0.402928495 |
| hsa-miR-940_R+1 | -1.16 | 0.406506851 |
| bta-miR-1246_L+1_1ss4TC | 0.39 | 0.407372673 |
| hsa-miR-28-3p | -0.06 | 0.411824657 |
| hsa-miR-130b-3p | -0.38 | 0.414918367 |
| hsa-miR-181c-3p_L-1R+1 | 0.36 | 0.419873828 |
| hsa-miR-200c-3p | 0.10 | 0.419957996 |
| bta-miR-1246_R+2_1ss2AC | 0.20 | 0.42072766 |
| hsa-miR-320c_R-1 | 0.23 | 0.422145711 |
| hsa-miR-320a-3p | 0.12 | 0.425412666 |
| hsa-miR-196a-5p | -0.14 | 0.425929179 |
| hsa-miR-1290_1ss13TG | -0.22 | 0.432590053 |
| ssc-miR-4332_L-1R-1_1ss2AC | 0.18 | 0.43308462 |
| mdo-miR-200a-3p_R+2 | 0.09 | 0.444197075 |
| hsa-miR-32-5p | -0.34 | 0.447142725 |
| hsa-miR-130a-5p | -0.91 | 0.447540339 |
| hsa-miR-450a-5p | 0.12 | 0.449527844 |
| hsa-miR-152-3p | -0.07 | 0.453988921 |
| hsa-miR-16-1-3p | -1.29 | 0.456826867 |
| hsa-miR-516a-5p_R-1 | -0.90 | 0.456890627 |
| hsa-miR-3177-3p | 1.10 | 0.458305066 |
| hsa-miR-576-3p | -1.02 | 0.459131267 |
| hsa-miR-758-3p_R-1 | 0.50 | 0.481983909 |
| hsa-miR-99b-3p_R+1 | 0.26 | 0.48694575 |
| hsa-miR-138-5p | 0.96 | 0.492216597 |
| hsa-miR-942-5p_L-2R+1 | 0.74 | 0.497852202 |
| hsa-miR-361-5p | -0.11 | 0.50141493 |
| hsa-miR-548k | -0.17 | 0.502974592 |
| hsa-miR-21-3p | -0.07 | 0.507352301 |
| hsa-miR-125a-3p_R-1 | -0.34 | 0.507587423 |
| hsa-miR-151a-5p | 0.11 | 0.507982388 |
| hsa-miR-382-3p_R+1 | -0.96 | 0.509986438 |
| hsa-miR-335-5p | -0.19 | 0.51040645 |
| hsa-miR-107_R-2 | 0.09 | 0.512298065 |
| hsa-miR-106b-5p | 0.12 | 0.514531358 |
| bta-miR-1246_R+2_1ss18GN | -0.88 | 0.515454647 |
| hsa-miR-29a-3p | -0.05 | 0.516841328 |
| hsa-mir-3960-p3_1ss16AT | -0.09 | 0.524461532 |
| hsa-miR-185-5p | -0.06 | 0.527622844 |
| hsa-let-7b-3p_1ss22CT | -0.41 | 0.528097113 |
| hsa-miR-769-5p | 0.06 | 0.536619871 |
| oga-miR-100_R+2 | 0.77 | 0.542873534 |
| hsa-miR-29c-3p | -0.10 | 0.546659599 |
| hsa-miR-3615_R+1 | -0.65 | 0.54701561 |
| eca-mir-8986a-p3 | 0.60 | 0.551552653 |
| hsa-miR-877-5p_R+3 | -0.27 | 0.557253288 |
| hsa-mir-3196-p5_1ss4GT | 0.81 | 0.563548899 |
| hsa-miR-331-5p_R-1 | -0.95 | 0.564650099 |
| hsa-let-7e-5p | 0.22 | 0.564954615 |
| hsa-mir-4454-p5 | 0.16 | 0.569199295 |
| hsa-miR-20a-5p | -0.03 | 0.572469784 |
| hsa-miR-378d_R-2 | 0.17 | 0.573082975 |
| mmu-mir-6240-p5_1ss20TC | -0.59 | 0.573580903 |
| hsa-miR-374b-3p | -0.88 | 0.58367213 |
| hsa-miR-30e-3p_1ss22CT | -0.06 | 0.585228233 |
| hsa-miR-125b-1-3p_R-1 | -0.40 | 0.587554802 |
| hsa-miR-301b-3p | -0.87 | 0.593759033 |
| hsa-miR-98-3p_1ss22CT | -0.31 | 0.598971982 |
| hsa-let-7g-5p | 0.04 | 0.599072225 |
| hsa-miR-181c-5p_R+2 | -0.13 | 0.601809478 |
| hsa-miR-10399-5p_R+1 | 0.33 | 0.60758075 |
| bta-miR-1246_L+4R+2 | 0.19 | 0.618003503 |
| hsa-miR-576-5p | -0.40 | 0.61980254 |
| hsa-miR-4326_R+4 | 0.19 | 0.62065489 |
| hsa-miR-132-3p | -0.19 | 0.627214229 |
| hsa-miR-21-5p_R+1 | 0.02 | 0.630273684 |
| hsa-miR-486-3p | -0.76 | 0.636315159 |
| hsa-miR-224-5p_L-1R-2 | 0.09 | 0.6393888 |
| mmu-miR-92a-3p_R+2 | 0.39 | 0.643898416 |
| bta-miR-1246_L+1R+1_2 | 0.31 | 0.655379196 |
| bta-miR-1246_L+1R+1_1 | 0.31 | 0.655379196 |
| hsa-miR-454-3p_R+1 | -0.13 | 0.655891039 |
| hsa-miR-25-3p | 0.05 | 0.662827139 |
| hsa-miR-651-5p | -0.07 | 0.665392437 |
| hsa-miR-4485-3p_L+1R+1 | 0.29 | 0.667466606 |
| cja-mir-1302-p5_1ss7GC | 0.92 | 0.678498602 |
| bta-miR-2424_L-2 | 0.99 | 0.679289889 |
| hsa-miR-93-5p | 0.02 | 0.681867185 |
| hsa-miR-379-5p | 0.23 | 0.685706906 |
| hsa-mir-10400-p5_2ss12GC19GA | 0.57 | 0.688139343 |
| hsa-miR-146b-5p_R+1 | -0.08 | 0.688780483 |
| hsa-miR-130a-3p | -0.06 | 0.696488644 |
| hsa-miR-205-5p | 0.08 | 0.697783104 |
| hsa-miR-532-5p | -0.06 | 0.698127106 |
| mmu-miR-92a-3p_R+1 | 0.13 | 0.699072702 |
| hsa-miR-1301-3p_R-1 | -0.14 | 0.706555042 |
| mdo-miR-34b-5p | -0.31 | 0.70755234 |
| hsa-miR-193b-3p | 0.03 | 0.707947838 |
| hsa-miR-23b-3p_R-1 | -0.03 | 0.71130483 |
| hsa-miR-2355-3p_L-2R+2 | -0.55 | 0.71208623 |
| hsa-miR-126-5p | -0.22 | 0.71625195 |
| hsa-miR-10a-3p_R-1 | 0.28 | 0.727016393 |
| bta-mir-2887-2-p5_1ss2CT | -0.52 | 0.727838222 |
| hsa-miR-103a-2-5p | -0.27 | 0.728279835 |
| hsa-miR-1271-5p | -0.17 | 0.729273454 |
| hsa-miR-454-5p | 0.30 | 0.732029812 |
| hsa-miR-193b-5p | -0.08 | 0.734434256 |
| hsa-miR-323a-3p | 0.75 | 0.741089098 |
| hsa-miR-23a-3p_R+1 | -0.04 | 0.742753937 |
| hsa-let-7i-3p | -0.13 | 0.750794368 |
| hsa-miR-340-5p | -0.07 | 0.753430606 |
| hsa-miR-378i_R+1_1ss9AT | -0.07 | 0.756006904 |
| hsa-miR-148a-5p | -0.29 | 0.757284543 |
| hsa-miR-24-2-5p_L+1R-1 | 0.12 | 0.757297723 |
| mmu-mir-8112-p5_1ss13CT | 0.25 | 0.761423122 |
| hsa-miR-134-5p | -0.47 | 0.774287562 |
| oga-miR-100_R+1 | -0.04 | 0.777783586 |
| mmu-mir-6236-p3 | -0.05 | 0.784636554 |
| hsa-miR-486-5p | -0.05 | 0.785265219 |
| hsa-miR-18a-3p | -0.27 | 0.786386372 |
| hsa-miR-548o-3p | -0.39 | 0.788430063 |
| hsa-miR-32-3p_R-1 | 0.34 | 0.789262147 |
| mmu-miR-146a-5p_R+1 | -0.14 | 0.790728962 |
| bta-miR-11987_L-2_1ss8TC | 0.04 | 0.790816149 |
| hsa-mir-4430-p3_1ss14AG | 0.56 | 0.799971024 |
| hsa-miR-487b-3p | 0.28 | 0.805561684 |
| hsa-miR-378d_1ss20AG | 0.04 | 0.806790541 |
| hsa-miR-378c_R-5 | 0.04 | 0.806790541 |
| rno-miR-1843b-3p | 0.50 | 0.819736332 |
| mmu-mir-5126-p5_1ss15CT_1 | 0.50 | 0.819736332 |
| mmu-mir-5126-p5_1ss15CT_2 | 0.50 | 0.819736332 |
| hsa-miR-328-3p | -0.10 | 0.820536474 |
| hsa-miR-1291_R-2 | 0.15 | 0.821085157 |
| rno-miR-1843b-5p_L+1R-2_1ss19AG | 0.08 | 0.822421597 |
| bta-miR-11987_L-2_1ss8TA | 0.04 | 0.823949022 |
| hsa-miR-654-3p_R-2 | -0.36 | 0.825906298 |
| cgr-miR-1285_L-6 | -0.04 | 0.832578141 |
| bta-mir-1246-p3_2ss5AC19AG | -0.20 | 0.833177273 |
| hsa-mir-663a-p3 | -0.37 | 0.836412259 |
| hsa-mir-663b-p3 | -0.37 | 0.836412259 |
| hsa-miR-378a-5p | -0.14 | 0.840150229 |
| hsa-miR-181b-5p_R+1 | -0.02 | 0.843290388 |
| hsa-miR-219a-5p_R+2 | -0.19 | 0.843892996 |
| hsa-miR-664a-3p | -0.07 | 0.844218754 |
| hsa-miR-652-3p_R+1 | 0.04 | 0.849230825 |
| hsa-miR-154-3p | -0.29 | 0.849406993 |
| hsa-miR-409-5p | -0.29 | 0.863313835 |
| bta-miR-11987_L-1R+1_1ss8TA | -0.12 | 0.864726249 |
| hsa-miR-122-5p_R-1 | -0.37 | 0.865012081 |
| hsa-miR-381-3p | -0.04 | 0.865224235 |
| hsa-miR-320b_R-2 | -0.04 | 0.865870059 |
| hsa-miR-19a-3p | -0.02 | 0.867948977 |
| hsa-let-7g-3p_R+1 | 0.12 | 0.871958345 |
| hsa-miR-339-3p | 0.12 | 0.875854747 |
| hsa-miR-190b-5p | -0.03 | 0.878751175 |
| hsa-miR-550a-3p | -0.10 | 0.891688207 |
| bta-mir-11987-p3_1ss6TA | -0.03 | 0.901475463 |
| hsa-mir-9902-1-p3_1ss13GC | -0.04 | 0.910155235 |
| hsa-miR-16-2-3p_L+1R-1 | -0.04 | 0.911498789 |
| hsa-miR-615-3p_R-1 | -0.04 | 0.915988735 |
| hsa-miR-181a-2-3p | 0.02 | 0.917913154 |
| hsa-miR-196b-5p | -0.02 | 0.920066731 |
| hsa-miR-146a-5p | -0.01 | 0.920067275 |
| hsa-miR-18a-5p | -0.04 | 0.922946264 |
| hsa-miR-10399-3p_L+1_1ss22CT | -0.09 | 0.925209028 |
| hsa-miR-200b-3p_R+1 | 0.01 | 0.927153399 |
| hsa-miR-22-5p | 0.03 | 0.929238797 |
| hsa-miR-27a-5p | 0.05 | 0.946183171 |
| hsa-miR-1910-5p | 0.14 | 0.948522277 |
| hsa-miR-191-5p | -0.01 | 0.948943447 |
| hsa-miR-339-5p_R-3 | -0.01 | 0.95587392 |
| mmu-mir-6240-p5_1ss21TC | 0.12 | 0.955986456 |
| hsa-miR-378a-3p | 0.00 | 0.959308884 |
| hsa-miR-423-3p | -0.01 | 0.960544183 |
| mmu-mir-5100-p3_1ss1AG | 0.01 | 0.964653577 |
| hsa-miR-92b-3p | -0.01 | 0.968957643 |
| pal-miR-9298-5p_R+2 | 0.05 | 0.969278333 |
| pal-mir-9298-p3 | -0.05 | 0.971741778 |
| bta-miR-378_R+2 | 0.03 | 0.98510709 |
| hsa-miR-590-5p | -0.01 | 0.986902661 |
| hsa-miR-660-5p_R+1 | 0.00 | 0.99602116 |

Ex: Exosomes secreted from non-infected OSCC cells

*Pg*.Ex: Exosomes secreted from *Pg*-infected OSCC cells

red, *p*<0.001; orange, *p*<0.01; blue, *p*<0.05; green, *p*<0.1
